# Tackling Allergic Airway Inflammation with Organic Sheet‐Like Nanoplatforms by Targeted Elimination of Epithelial Small Extracellular Vesicles

**DOI:** 10.1002/advs.202504197

**Published:** 2025-09-26

**Authors:** Zhaoxu Tu, Junyan Lin, Zhixin Li, Yuefei Zhu, Changyi Xu, Zihan Qiu, Qiumin Wang, Yang Ye, Yihui Wen, Jian Li, Kam W. Leong, Weiping Wen

**Affiliations:** ^1^ Department of Otolaryngology The Sixth Affiliated Hospital Sun Yat‐sen University Guangzhou Guangdong 510655 China; ^2^ Department of Otolaryngology The First Affiliated Hospital Sun Yat‐sen University Guangzhou Guangdong 510080 China; ^3^ Department of Biomedical Engineering Columbia University New York NY 10027 USA; ^4^ Biomedical Innovation Center The Sixth Affiliated Hospital Sun Yat‐sen University Guangzhou Guangdong 510655 China; ^5^ Key Laboratory of Human Microbiome and Chronic Diseases (Sun Yat‐sen University) Ministry of Education Guangzhou 510655 China

**Keywords:** allergic airway inflammation, dendritic cells, eosinophil extracellular trap, epithelium, organic nanosheets, small extracellular vesicles

## Abstract

Allergic airway inflammatory disorders, such as allergic rhinitis (AR) and asthma, affect the health of more than one billion people worldwide, yet therapeutic outcomes remain unsatisfactory. 2D nanomaterials are extensively adopted in biomedical research, but their inorganic components often limit clinical applications. To address these challenges, functionalized “inorganic‐free” nanosheets PNS_E_ are developed as a potential strategy for alleviating allergic inflammation via targeted elimination of epithelial small extracellular vesicles (sEVs). PNS_E_ are prepared using template‐based synthesis technology and modified with epidermal growth factor receptor aptamers, which exhibit low cytotoxicity, mild protein adsorption, and potent epithelial sEVs binding efficacy. PNS_E_ suppresses sEVs‐triggered stimulator of interferon genes activation, alleviating the dendritic cell maturation and eosinophil extracellular trap formation in vitro. In addition, PNS_E_ displays exceptional biocompatibility, preferential airway localization, and robust modulation for allergic airway inflammation in vivo. Transcriptome analysis and multi‐channel flow cytometry of airway tissues further confirm the alleviation of dysregulated airway inflammation in house dust mite‐stimulated animal models. These results highlight the pivotal feature of the organic sheet‐like nanoplatforms for targeted clearance of epithelial sEVs, which can be exploited as a nanomedicine for the treatment of allergic airway inflammation and also other allergic disorders.

## Graphical abstract

In this work, “inorganic‐free” nanosheets, named PNS_E_ are developed using template‐based synthesis technology. The functionalized organic nanosheets exhibit low cytotoxicity, mild protein adsorption, and potent binding efficacy to epithelial sEVs. Furthermore, PNS_E_ suppresses sEV‐triggered sEVs‐triggered stimulator of interferon gene activation, alleviating the DC maturation and eosinophil extracellular trap formation in vitro, and efficiently modulates allergic airway inflammation in house dust mite‐stimulated model mice.

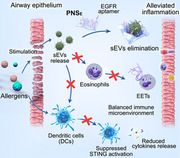

## 1 Introduction

Allergic airway diseases (AAD), including allergic rhinitis (AR) and bronchial asthma, represent a significant global public health issue, affecting over 20% of the world's population.^[^ ^1^, ^2^, ^3^ ^]^ The respiratory allergic inflammation is predominantly driven by type 2 immune responses characterized by immune cell infiltration, overdone mucus secretion, and airway hyperresponsiveness.^[^ ^3^, ^4^, ^5^ ^]^ The epithelium of the airway mucosa is the first place where the human body comes into contact with inhaled allergens, and allergen stimulation can damage the airway epithelium.^[^ ^5^, ^6^, ^7^ ^]^ Small extracellular vesicles (sEVs) are tiny vesicles secreted by cells under normal and pathological conditions, mediating intercellular communication between distant cells.^[^ ^8^, ^9^, ^10^ ^]^ Recent studies have shown that epithelial‐derived sEVs are involved in various inflammatory diseases and can amplify downstream inflammatory responses by activating key signaling pathways.^[^ ^10^, ^11^, ^12^ ^]^ In our preliminary experiments, we found that levels of sEVs and double‐strand DNA (dsDNA) in the nasal secretions of AR patients were significantly higher than those in healthy controls, with sEV levels positively correlated with the severity of inflammation (Figure 2A–D).

The therapeutic efficacy of current clinical treatments for AAD, such as corticosteroids and antihistamines, remains limited in some patients, and none specifically target epithelial sEVs implicated in allergic inflammation.^[^ ^6^, ^13^, ^14^ ^]^ Over the past few decades, advances in nanotechnology have offered novel therapeutic strategies for addressing refractory airway disorders.^[^ ^15^, ^16^, ^17^, ^18^, ^19^ ^]^ Due to their flexible, crimpable, and planar nanostructure, functional nanosheets provide greater adaptability to the shape and structure of sEVs compared to their spherical counterparts.^[^ ^19^, ^20^ ^]^ Thus, the removal of epithelial sEVs using 2D nanoplatforms could be a promising therapeutic strategy for allergic airway inflammation. Planar‐structured nanosheets, such as graphene, black phosphorus, and transition‐metal dichalcogenides, have been extensively explored in biomedical research; however, their inorganic composition often hinders clinical translation.^[^ ^19^, ^20^, ^21^, ^22^ ^]^ In contrast, organic polymers typically exhibit superior biocompatibility, minimizing the risk of immune responses and rapid clearance in vivo.^[^ ^23^, ^24^ ^]^

Building upon this, we tried to design an “inorganic‐free” 2D nanoplatform by degrading the inorganic backbone to construct polyglycerol‐amine (PG) nanosheets.^[^ ^23^ ^]^ PG was selected in this study due to its excellent biocompatibility, protein resistance, tunable blood circulation, and chemical stability.^[^ ^24^, ^25^ ^]^ In addition, aptamers are functional oligonucleotides that are able to specifically bind to their targets with high affinity due to their unique tertiary structures.^[^ ^15^ ^]^ The epidermal growth factor receptor (EGFR) enrichment expression was confirmed on epithelial‐derived sEVs,^[^ ^26^ ^]^ suggesting that the modification of EGFR aptamers (AptE) onto the organic nanosheets is promising to optimize the targeted binding between nanosheets and epithelial sEVs. In addition to epithelial sEVs, persistent reactive oxygen and nitrogen species (RONS) generation is known to contribute to allergic airway inflammation.^[^ ^27^ ^]^ The residual thiol groups present in the organic nanosheets are promising to exhibit anti‐oxidant properties by neutralizing RONS through electron donation, converting them into more stable and less reactive molecules^[^ ^27^ ^]^ which is also beneficial to mitigate the allergen‐stimulated dysregulated inflammatory response.

**Figure 1:**
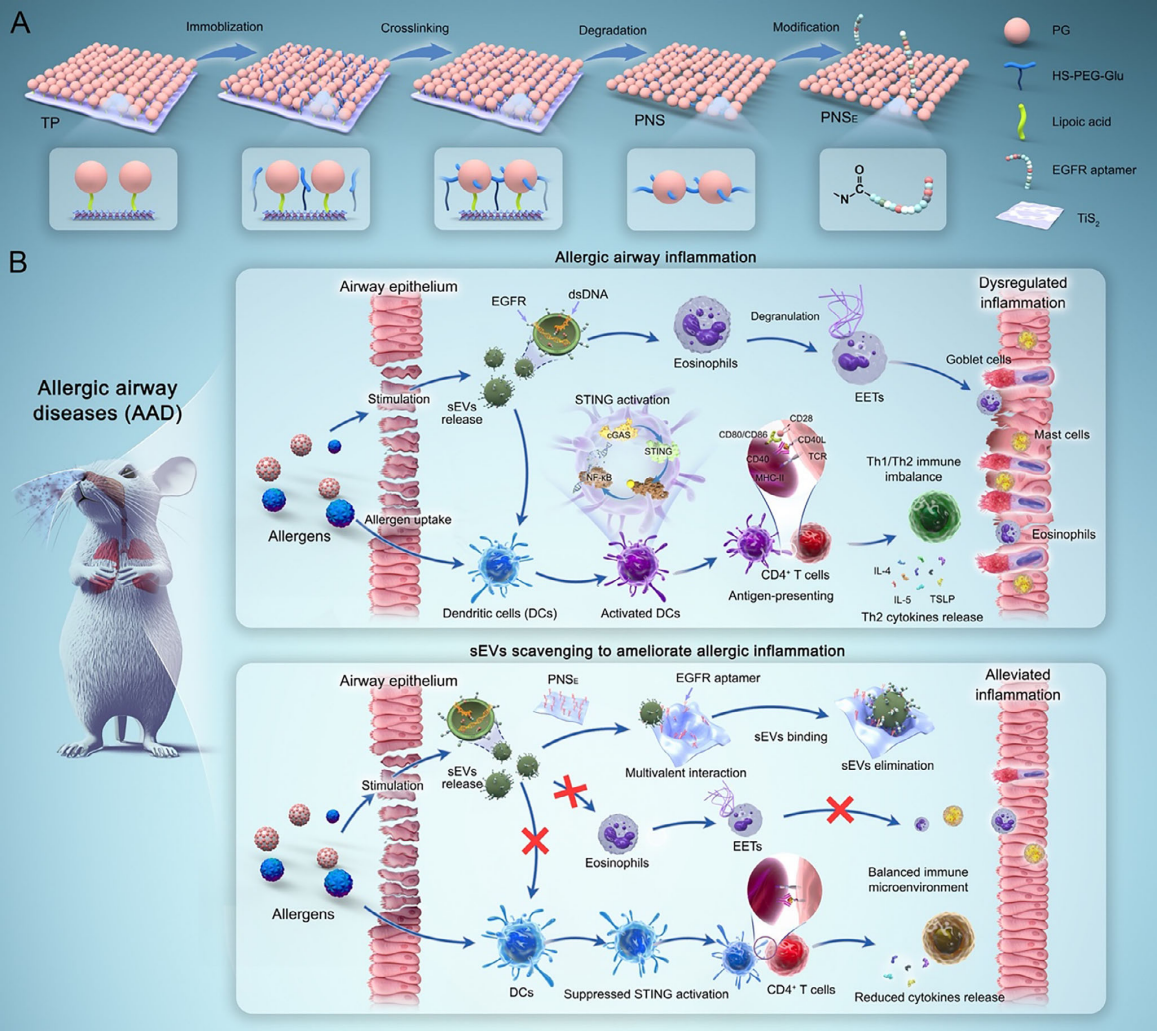
Schematic illustration of “inorganic‐free” nanosheet preparation and anti‐inflammation application. A) The synthesis routes of PNS_E_ via sequential immobilization, crosslinking, degradation, and modification. B) Schematic of the allergic airway inflammation induced by epithelial sEVs and tackling allergic airway inflammatory disorders in model mice with PNS_E_.

In this work, we first observed elevated levels of sEVs and dsDNA in the nasal secretions of AR patients, and subsequently elucidated the role of epithelial sEVs in the pathology of allergic airway inflammation. A mouse model of allergic airway inflammation was established, revealing that suppression of allergen‐stimulated sEVs could alleviate the inflammatory response. To target epithelial sEVs, PG‐based “inorganic‐free” nanosheets (PNS) were designed, and AptE was modified to construct PNS_E_ for epithelial sEVs elimination (**Figure** **1**). The sEVs binding capacity, biocompatibility, protein adsorption, and RONS reduction capacities were carefully examined. Subsequently, we evaluated whether PNS_E_ could suppress the epithelial sEVs‐induced dendritic cell (DC) activation and eosinophil extracellular trap (EET) formation. Additionally, the accumulation of PNS_E_ in the inflamed airway tissues of model mice following intranasal administration was comprehensively assessed. Finally, we investigated the anti‐inflammatory effects of PNS_E_ in both upper and lower airways by measuring cytokine levels, eosinophil cationic protein (ECP) expression, and goblet cell hyperplasia after PNS_E_ administration. RNA sequencing (RNA‐seq) transcriptome analysis of inflamed airway tissues was performed to gain detailed insights into the therapeutic mechanisms and efficacy.

## 2 Results

### 2.1 Analysis of sEVs Number and dsDNA Level in Nasal Secretions of AR Patients

**Figure 2:**
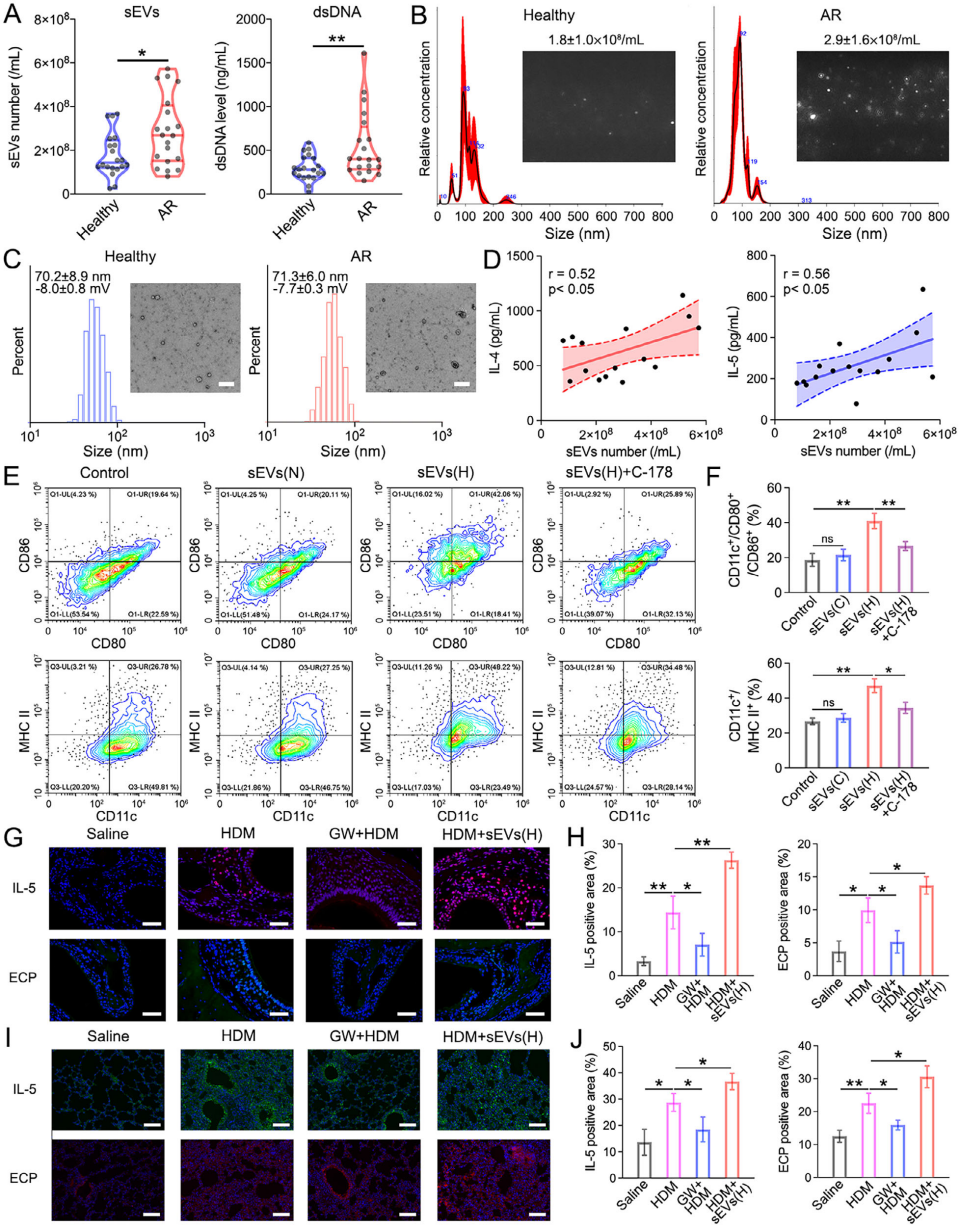
sEVs and dsDNA in allergic airway inflammation. A) The sEVs number and dsDNA level in nasal secretions of healthy volunteers and AR patients. Data represent the mean ± S.D. (*n* = 20, Student's *t*‐test, *p < 0.05, and **p < 0.01). B) The NTA results of sEVs isolated from nasal secretions. C) The DLS results and TEM images of sEVs isolated from nasal secretions. Scale bars: 200 nm. D) The correlations between IL‐4 and IL‐5 with relative sEVs number in nasal secretions from AR patients. E) Representative CD11c^+^/CD80^+^/CD86^+^ or CD11c^+^/MHC II^+^ in the BMDCs treated with sEVs(N), sEVs(H), and sEVs(H)+C‐178. F) Quantification of the flow cytometry results of BMDCs. Data represent the mean ± S.D. (*n* = 3, one‐way ANOVA, ns represents not significant, *p < 0.05, **p < 0.01). G) Representative IL‐5 or ECP immunostaining images of the nasal mucosa from experimental mice. Scale bars: 50 µm. H) Quantification of the IL‐5, or ECP positive area in slices of nasal mucosa. I) Representative IL‐5 or ECP immunostaining images of the lungs from experimental mice. Scale bars: 50 µm. J) Quantification of the IL‐5, or ECP positive area in slices of lungs. Data represent the mean ± S.D. (*n* = 6, one‐way ANOVA, *p < 0.05, **p < 0.01).

A total of 20 AR patients and 20 healthy volunteers were recruited for this project, and their nasal secretions were collected. sEVs were isolated from the nasal secretions using sequential centrifugation at 100 × g (10 min), 2000 × g (10 min), 10 000 × g (30 min), and 100 000 × g (70 min), respectively.^[^ ^28^ ^]^ According to representative images and statistical outcomes from nanoparticle tracking analysis (NTA), the isolated sEVs had a size of ≈100 nm in both the healthy and AR groups (**Figure** **2**A). Notably, the number of sEVs was significantly higher in nasal secretions from AR patients compared to healthy volunteers (2.9 ± 1.6 × 10^8^ vs 1.8 ± 1 × 10^8^ particles mL^−1^, 97 ± 7.9 and 107.7 ± 13.3 nm), and the concentration of double‐stranded DNA (dsDNA) detected by pico‐green assay also displayed a similar trend (551 ± 383 vs 297 ± 145 ng mL^−1^) (Figure 2B).^[29]^ To validate the NTA results, the sEVs were characterized by dynamic light scattering (DLS) and transmission electron microscopy (TEM). The zeta potential of sEVs was −8 ± 0.8 and −7.7 ± 0.3 mV, and TEM images demonstrated the nanoscale spherical morphology of extracted sEVs. The size calculated by TEM was ≈70 nm, which is smaller than NTA and DLS, attributed to the hydrodynamic effect (Figure 2C).^[^ ^19^ ^]^

To further analyze the correlation between sEVs and allergic inflammation, we quantitatively determined interleukin‐4 (IL‐4) and interleukin‐5 (IL‐5) levels, characteristic cytokines in allergic responses, in the nasal secretions. Significant positive associations between sEVs number and IL‐4 level (*r* = 0.52, *p* < 0.05) or IL‐5 level (*r* = 0.56, *p* < 0.05) were identified (Figure 2D). Consistent with sEVs, the dsDNA concentration was also found to be positively correlated with IL‐4 level or IL‐5 level, respectively (Figure S1, Supporting Information). These results collectively revealed allergen stimulation in the airway epithelial is possible to elicit elevated sEVs and dsDNA generation, and they were closely bound up with the allergic inflammation.

### 2.2 Allergen‐Elicited sEVs Exacerbated Airway Inflammation

As epithelial serve as the first barrier against inhaled allergens in the airway, primary mouse epithelial cells were incubated with house dust mite (HDM), a common allergen, to simulate the interaction between allergen and airway epithelial.^[^ ^30^, ^31^ ^]^ HDM sensitization caused more sEVs generation, and sEVs(H) and sEVs(N) were isolated from the medium of HDM‐treated cells and the medium of untreated cells, respectively (Figure S2, Supporting Information). Given that the maturation and antigen‐presenting capacity of DCs play pivotal roles in allergic airway inflammatory disorders, bone marrow‐derived DCs (BMDCs) were isolated from the mice. Subsequently, BMDCs were incubated with the isolated sEVs, and the results of flow cytometry displayed sEVs(H) elicited obviously higher percent of CD11c^+^/CD86^+^/CD80^+^ cells (41 ± 4.4%), compared to the control group (18.7 ± 3.6%) and also the sEVs(N) group (21.5 ± 3.3%) (Figure 2E,F). A similar trend was also observed in the CD11c^+^/CD40^+^ cells (Figure S3, Supporting Information), indicating a higher maturation degree of BMDCs following sEVs(H) treatment. In addition, the proportion of CD11c^+^/MHC II^+^ cells also increased after incubation with sEVs(H) compared to the control and sEVs(N) group (47.1 ± 4% vs 26.7 ± 1.9% vs 28.7 ± 2.5%), indicating the elevated antigen‐presenting ability (Figure 2E,F). Since dsDNA was extensively recognized to activate the cGAS‐STING pathway, C‐178, an STING inhibitor, was added to the BMDCs prior to sEVs(H) treatment.^[^ ^32^, ^33^ ^]^ It was found that C‐178 suppressed the sEVs‐induced BMDCs maturation and declined the antigen‐presenting capacity, pointing out that the cGAS‐STING pathway is critical for the DC activation. In order to clarify the pivotal role of dsDNA, sEVs(H) were pretreated with DNase, Sonication, or Sonication+DNase before incubation with the HEK‐STING cells. The outcomes of the Quanti‐blue assay displayed only Sonication+DNase treatment could modulate the sEVs(H)‐triggered STING activation (Figure S4, Supporting Information), confirming dsDNA was incorporated inside sEVs and could only be degraded by DNase after disrupting the sEVs membrane by sonication.

In addition to the overactivation of DCs, eosinophils were reported to be involved in allergic airway diseases, especially in patients with type 2 inflammation.^[^ ^34^, ^35^ ^]^ RPMI2650 cells (human nasal epithelial cell line) were incubated with HDM, and sEVs were isolated from the culture medium using the same protocol (Figure S5, Supporting Information). Afterward, eosinophils were isolated from the peripheral blood of AR patients and were employed to investigate EET formation.^[^ ^34^, ^35^ ^]^ It was observed in confocal laser scanning microscopy (CLSM) images that the ECP‐positive area was significantly larger in sEVs(H)‐treated eosinophils (13.3 ± 2.8%) than in the control group (1.7 ± 1.2%) and sEVs(N) group (2.9 ± 0.5%) (Figure S6, Supporting Information). Moreover, C‐178 pre‐treatment suppressed ≈50% of the EET formation, indicating the sEVs‐induced EET formation was also highly bound up with the cGAS‐STING pathway. The above‐mentioned experiments collectively demonstrated the critical role of allergen‐stimulated sEVs in DC maturation and EET formation, indicating sEVs could be a potential therapeutic target for allergic airway inflammatory disorders.

To further investigate the potential of modulating allergic airway inflammation by suppressing allergen‐induced sEV generation, we established a mouse model via HDM instillation (Figure S7A, Supporting Information).^[^ ^31^, ^35^ ^]^ GW4869, a neutral sphingomyelinase inhibitor that blocks the release of sEVs,^[^ ^16^, ^36^ ^]^ was administered to one group of HDM‐treated mice. In another group, additional sEVs were instilled into the experimental mice. Bronchoalveolar lavage fluid (BALF) was gathered 24 h after the last treatment, and it was found that the sEVs number, as well as the dsDNA level, was largely decreased in BALF due to the GW4869 treatment (Figure S7B, Supporting Information). Whereafter, maxillary bones (MBs) and lungs were collected from the experimental mice, and IL‐5 and ECP immunostaining were utilized after decalcification and fixation of the organs. As depicted in Figure 2G,H, there was an increase in IL‐5 and ECP positive areas—by 4.4‐fold and 2.7‐fold, respectively—in the nasal mucosa of HDM‐instilled mice. Treatment with GW4869 effectively alleviated the inflammatory response, whereas the instillation of additional sEVs further exacerbated nasal inflammation, with IL‐5 and ECP levels rising by 1.8‐fold and 1.4‐fold compared to the HDM group, respectively. In addition to the upper‐airway, the analysis of lung slices also revealed that GW4869 or additional sEVs treatment could ameliorate or boost the lower‐airway inflammation, respectively (Figure 2I,J). Altogether, these results confirmed the dominant role of allergen‐induced sEVs in allergic airway inflammation, suggesting suppression of sEVs generation could be a potential therapeutic strategy. However, GW4869 was unsuitable for clinical application owing to its serious toxicity and unavoidable side effects.^[^ ^16^, ^36^ ^]^

### 2.3 Synthesis and Characterization of Organic Sheet‐Like Nanoplatforms

According to the above results, developing “inorganic‐free” PG nanosheets by removing the inorganic backbone presents an effective strategy for creating functional organic sheet‐like nanoplatforms (Figure 1). To achieve this, TiS_2_ monolayer nanosheets were synthesized using a lithium‐ion intercalation method.^[^ ^37^, ^38^ ^]^ The PG was then attached to the nanosheets via amidation, forming PG‐covered TiS_2_ (TP). Next, crosslinkers were immobilized onto the backbone before crosslinking the dendritic PG molecules locally. Finally, horseradish peroxidase (HRP) was used to degrade the TiS_2_ template,^[^ ^39^ ^]^ resulting in PNS. Western blot (WB) analysis revealed the overexpression of EGFR on epithelial sEVs (Figure S8, Supporting Information). Consequently, Apt_E_ was conjugated to the nanosheets (PNS_E_) to enhance binding affinity to epithelial sEVs (Figures S9,S10, Supporting Information).

**Figure 3:**
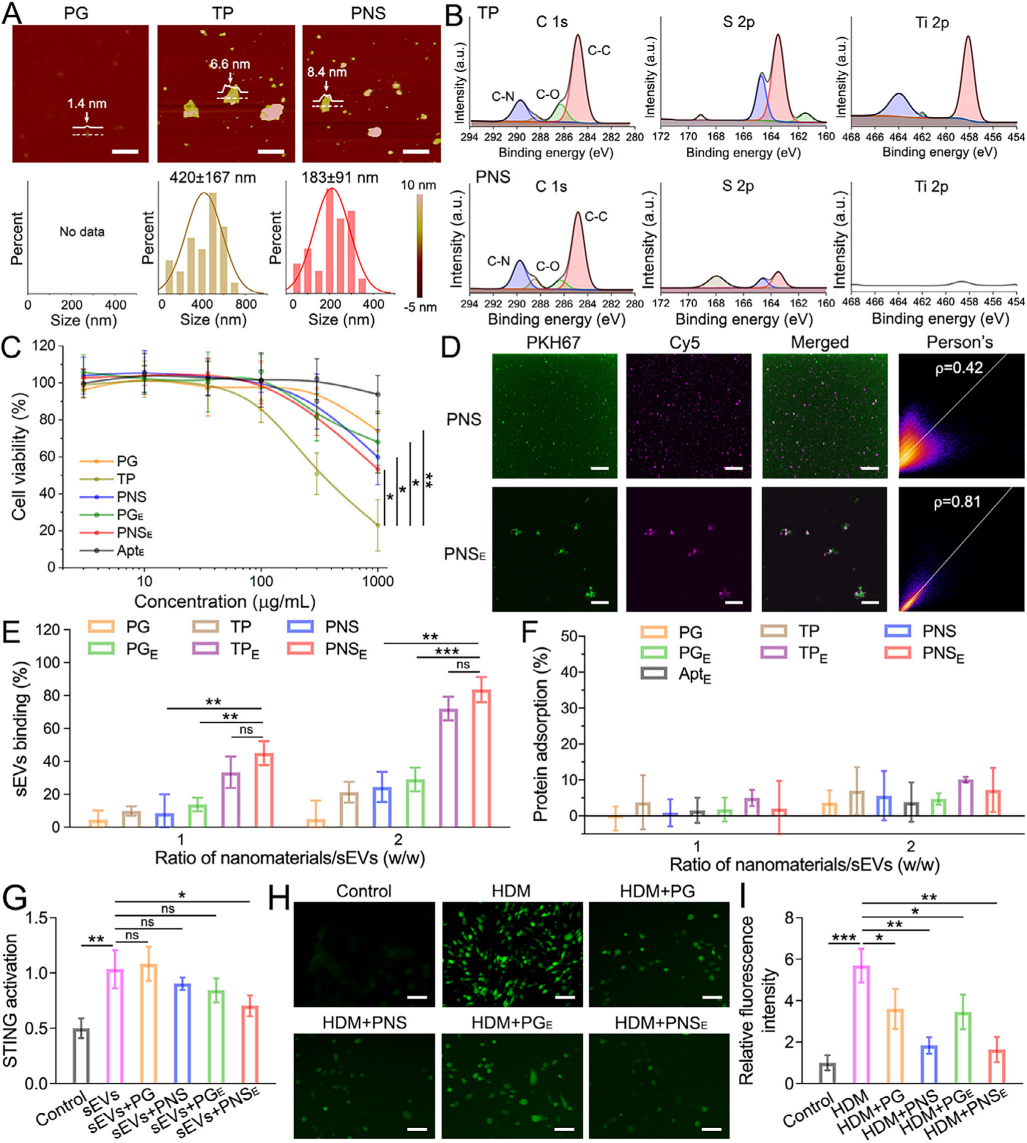
Synthesis and characterization of organic nanosheets. A) AFM images and corresponding size distribution of PG, TP, and PNS. Scale bars: 200 nm. B) High‐resolution XPS mapping (C 1s, S 1s, and Ti 1s) of TP and PNS. C) Viability of RPMI2650 cells treated for 24 h with various concentrations of PG, TP, PNS, PG_E_, PNS_E_, and Apt_E_. D) The CLSM images of PKH67‐labeled sEVs incubated with Cy5‐labeled PNS and PNS_E_ in PBS. Scale bars: 1 µm. Pearson's r analysis for the colocalization of green and red colors was applied. E) Quantitative sEVs‐binding capacities of PG, TP, PNS, PG_E_, TP_E_, and PNS_E_ with different ratios. F) Protein adsorption ability of PG, TP, PNS, PG_E_, TP_E_, PNS_E_, and Apt_E_ in the presence of BSA (10%). G) sEVs‐induced STING activation in HEK‐STING cells after incubation with PG, PNS, PG_E_, and PNS_E_. H) The CLSM images of DCFH fluorescence in HDM‐treated RPMI2650 cells incubated with PG, PNS, PG_E_, and PNS_E_ in PBS. Scale bars: 50 µm. I) Quantitative DCFH fluorescence intensities in CLSM images. Data represent mean ± S.D. (*n* = 3; one‐way ANOVA, ns represents not significant, *p < 0.05, **p < 0.01, ***p < 0.001).

Both TP and PNS retained a sheet‐like morphology, with average sizes of 420 ± 167 and 183 ± 91 nm, respectively, as determined by atomic force microscopy (AFM) (**Figure** **3**A). In contrast, PG molecules with a size less than 10 nm were barely detected in the AFM measurement, confirming the successful synthesis of TP and PNS. The measured heights were ≈6.6 nm for TP and 8.4 nm for PNS, indicating that PG crosslinking increased the size and height of the nanosheets. DLS results showed that the sizes of TP, PNS, TP_E_, and PNS_E_ were slightly smaller compared to AFM measurements, likely due to the folding properties of the 2D nanostructures (Figure S11, Supporting Information).^[^ ^19^, ^20^, ^21^ ^]^ UV–vis spectroscopy confirmed the complete degradation of TiS_2_ during the synthesis of the organic nanosheets, as indicated by the altered absorbance properties of PNS compared to TP (Figure S12, Supporting Information). The degradation was further validated by high‐resolution X‐ray photoelectron spectroscopy (XPS),^[^ ^40^ ^]^ where the disappearance of the Ti 2p peak in PNS confirmed the successful removal of TiS_2_ (Figure 3B). In contrast, XPS mapping of TP revealed the presence and distribution of C, S, O, and Ti elements (Figure 3B; Figure S13, Supporting Information).

Given the potential of fully organic nanosheets to improve biocompatibility, we evaluated their cytotoxicities. RPMI2650 cells were treated by PG, TP, PNS, PG_E_, PNS_E_, and Apt_E_ with a series of concentrations (3–1000 µg mL^−1^) for 24 h (Figure 3C). As expected, TP exhibited relatively lower biocompatibility than other groups, owing to the TiS_2_ backbone.^[^ ^41^, ^42^ ^]^ The viability of cells treated with PNS and PNS_E_ remained ≈60% even at 1000 µg mL^−1^, indicating slight cytotoxicity of “inorganic‐free” nanosheets. The difference in viability is more conspicuous for the experimental cells treated with nanomaterials for 48 h (Figure S14, Supporting Information). All of these PG‐based nanomaterials displayed mild unspecific adsorption, which is essential for the in vivo sEVs scavenging application.

### 2.4 sEVs Binding and Antioxidant Capacity of PNS_E_

The binding capacity of PNS and PNS_E_ to epithelial sEVs was determined by co‐incubating PKH67‐labeled sEVs (green) with Cy5‐labeled nanosheets (magenta).^[^ ^19^ ^]^ Substantial colocalization of sEVs with PNS_E_ was observed in the CLSM images, while the green fluorescence barely overlapped with the magenta fluorescence in the PNS group, as indicated by Pearson's r values (Figure 3D). Additionally, the quantitative binding efficacy of epithelial sEVs was measured at different ratios (w/w = 1 or 2) of nanomaterials to sEVs (Figure 3E). Apt_E_‐modified nanosheets (PNS_E_ and TP_E_) exhibited the highest binding efficiency (≈80%), indicating the targeting ligands and 2D geometry of nanoplatforms allow for better access to the sEVs.^[^ ^19^ ^]^ The protein resistance of PG was also epitomized in protein adsorption results of organic nanosheets (Figure 3F),^[^ ^24^, ^25^ ^]^ which is beneficial for targeted removal of epithelial sEVs in physiological conditions. To assess whether the sEV elimination approach could mitigate STING pathway activation triggered by sEVs, HEK‐STING cells were treated with sEVs alone and in combination with PG, PNS, PG_E_, and PNS_E_. While allergen‐induced epithelial sEVs strongly boosted the STING pathway, PNS_E_ modulated this inflammatory response (to ≈60%) by sEVs binding (Figure 3G). More importantly, the STING activation in PNS_E_ groups is much lesser than in PG, PNS, and PG_E_ groups, demonstrating the potential of PNS_E_ to relieve dysregulated immune responses by targeting epithelial sEVs.

Beyond sEVs, elevated reactive oxygen and RONS are also characteristic of inflamed tissues, and reducing RONS can help alleviate inflammation.^[^ ^43^, ^44^ ^]^ The results of 2,2‐Diphenyl‐1‐picrylhydrazyl (DPPH) and hydroxyl radicals (OH^−^) reduction assays displayed the robust antioxidant activities of PNS and PNS_E_ (Figure S15, Supporting Information).^[^ ^45^ ^]^ As shown in Figure 3H, HDM‐treated RPMI2650 cells were incubated with PG, PNS, PG_E_, and PNS_E_ before dichlorodihydrofluorescein diacetate (DCFH‐DA) was added.^[^ ^46^ ^]^ Afterward, the intracellular reactive species level was observed by CLSM (488 nm/525 nm), and the quantitative fluorescent intensities in PNS and PNS_E_‐treated cells were considerably lower than those in the HDM‐treated group. More interestingly, PNS and PNS_E_ treatments exhibited a more substantial decrease in reactive species compared to PG and PG_E_ groups (Figure 3H,I). In summary, it underscores the potential of PNS and PNS_E_ to mitigate oxidative stress in inflammatory conditions of airway disorders.

### 2.5 PNS_E_ Suppressed DC Activation and EET Formation

**Figure 4:**
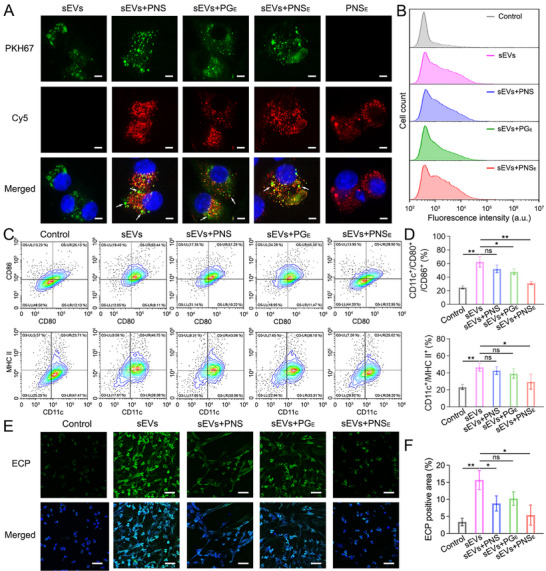
Suppression of DC maturation and EET formation by organic nanosheets. A) CLSM images of BMDCs after 24 h incubation with sEVs, sEVs+PNS, sEVs+PG_E_, and sEVs+PNS_E_. sEVs were labeled with PKH67; PNS, PG_E_, and PNS_E_ were labeled with Cy5. Scale bars, 5 µm. B) The sEVs uptake efficacy of BMDCs after 24 h incubation with sEVs, sEVs+PNS, sEVs+PG_E_, and sEVs+PNS_E_. sEVs were labeled with PKH67. C) Representative CD11c^+^/CD80^+^/CD86^+^ or CD11c^+^/MHC II^+^ in the BMDCs treated with sEVs, sEVs+PNS, sEVs+PG_E_, and sEVs+PNS_E_. D) Quantification of the BMDCs maturation after different treatments. E) Representative ECP immunostaining images of eosinophils treated with sEVs, sEVs+PNS, sEVs+PG_E_, and sEVs+PNS_E_. Scale bars: 20 µm. F) Quantification of the ECP positive area in CLSM images. Data represent mean ± S.D. (*n* = 3; one‐way ANOVA, ns represents not significant, *p < 0.05, **p < 0.01).

Since STING receptors are located on the endoplasmic reticulum (ER) of cells,^[^ ^47^ ^]^ it is important to investigate the cellular uptake and intracellular colocalization of epithelial‐derived sEVs after interaction with nanosheets. PKH67‐labeled sEVs (green) and Cy5‐labeled PNS, PG_E_, and PNS_E_ (red) were incubated with BMDCs for 24 h. CLSM images showed minimal overlap between the green fluorescence of sEVs and the red fluorescence of PNS and PG_E_. In contrast, cells treated with sEVs and PNS_E_ exhibited significant colocalization, suggesting that PNS_E_ maintained strong binding even in the complex intracellular environment (**Figure** **4**A). Flow cytometry analysis further indicated that PNS, PG_E_, and PNS_E_ treatment did not significantly reduce the internalization efficiency of sEVs (Figure 4B). The relative mean fluorescence intensity (MFI) value in the sEVs group, sEVs+PNS group, sEVs+PG_E_ group, and sEVs+PNS_E_ group was 2.4 ± 1, 1.9 ± 0.8, 2 ± 0.2, and 2.2 ± 0.3, respectively. Based on these observations, we explored whether PNS_E_ can modulate the allergic inflammatory response triggered by epithelial sEVs.

As allergen‐induced epithelial sEVs were tightly related to the allergic airway inflammation, and PNS_E_ displayed robust sEVs binding capacity, it was quite fascinating to test whether PNS_E_ could suppress the sEVs‐triggered pro‐inflammatory immune cascade. BMDCs were isolated from healthy mice and incubated with sEVs, sEVs+PNS, sEVs+PG_E_, and sEVs+PNS_E_ for 24 h. Subsequently, the cells were collected, and the maturation status and antigen‐presenting capacity were analyzed by flow cytometry (CD11c, CD40, CD80, CD86, and MHC II).^[^ ^48^, ^49^ ^]^ The results showed that PNS_E_ treatment markedly declined the proportion of CD11c^+^/CD80^+^/CD86^+^ and CD11c^+^/CD40^+^ cells compared to the sEVs group (31.3 ± 2.3% vs 62.6 ± 7.8%, and 25.1 ± 3.8% vs 48.3 ± 4.9%), indicating that DC activation was alleviated (Figure 4C,D; Figure S16, Supporting Information). In addition, the anti‐presenting capacity of sEVs‐incubated BMDCs was also mitigated after PNS_E_ treatment, confirmed by the decrease of CD11c^+^/MHC II^+^ cells (29.6 ± 8.9% vs 46.7 ± 4.6%). PNS and PG_E_ treatments also suppressed the DC maturation and antigen‐presenting capacity (Figure 4C,D). Nevertheless, the inhibition effects were insignificant and much less than those in the PNS_E_ group.

Previous results showed that allergen‐induced epithelial sEVs could sensitize the degranulation of eosinophils and the generation of EETs, contributing to allergic airway inflammation.^[^ ^25^, ^38^ ^]^ The statistical analysis of immunostaining images revealed that the elevated ECP level in sEVs‐treated eosinophils was reduced after PNS_E_ treatment (from 15.7 ± 2.8% to 5.4 ± 3%), and the reduction was more pronounced than that in PNS and PG_E_ groups (8.8 ± 2.2% and 10.2 ± 2%) (Figure 4E,F). These consequences jointly unraveled the anti‐inflammation properties of PNS_E_ via the elimination of epithelial sEVs after allergen stimulation. Furthermore, the importance of nanoscale 2D geometry and Apt_E_ was also highlighted in the development of nanomedicine for allergic airway inflammation.

### 2.6 Biodistribution of Nanosheets after Administration

Based on the sterling in vitro inflammation modulation effect of PNS_E_ by targeting epithelial sEVs, we evaluated the in vivo safety and therapeutic efficacy of these nanosheets for allergic airway inflammation in a mouse model. First, the safety profiles of PNS, PG_E_, TP_E_, and PNS_E_ with large dosages (10 mg per time and four times in total) in healthy mice were determined. On the 14th or 28th day after intranasal instillation, the MBs were isolated and decalcified and finally fixed with other major organs (hearts, livers, spleen, lungs, and kidneys) for hematoxylin−eosin (H&E) staining.^[^ ^25^, ^38^ ^]^ Significant tissue injuries and inflammatory cell infiltration were observed in the livers and lungs of TP_E_‐treated mice, indicating the evident in vivo toxicity due to the inorganic TiS_2_ backbone (Figures S17,S18, Supporting Information). Fortunately, superior biocompatibility was confirmed for PNS, PG_E_, and PNS_E_, as negligible injuries were found in the experimental mice compared to the sham mice treated with saline. Further validation through hepatic and renal function assays in mouse serum confirmed that eliminating the inorganic backbone improved the biosafety of functional nanosheets (Figures S19,S20, Supporting Information), supporting their potential for clinical application. As a result, PNS, PG_E_, and PNS_E_ were selected for subsequent anti‐inflammatory studies.

**Figure 5:**
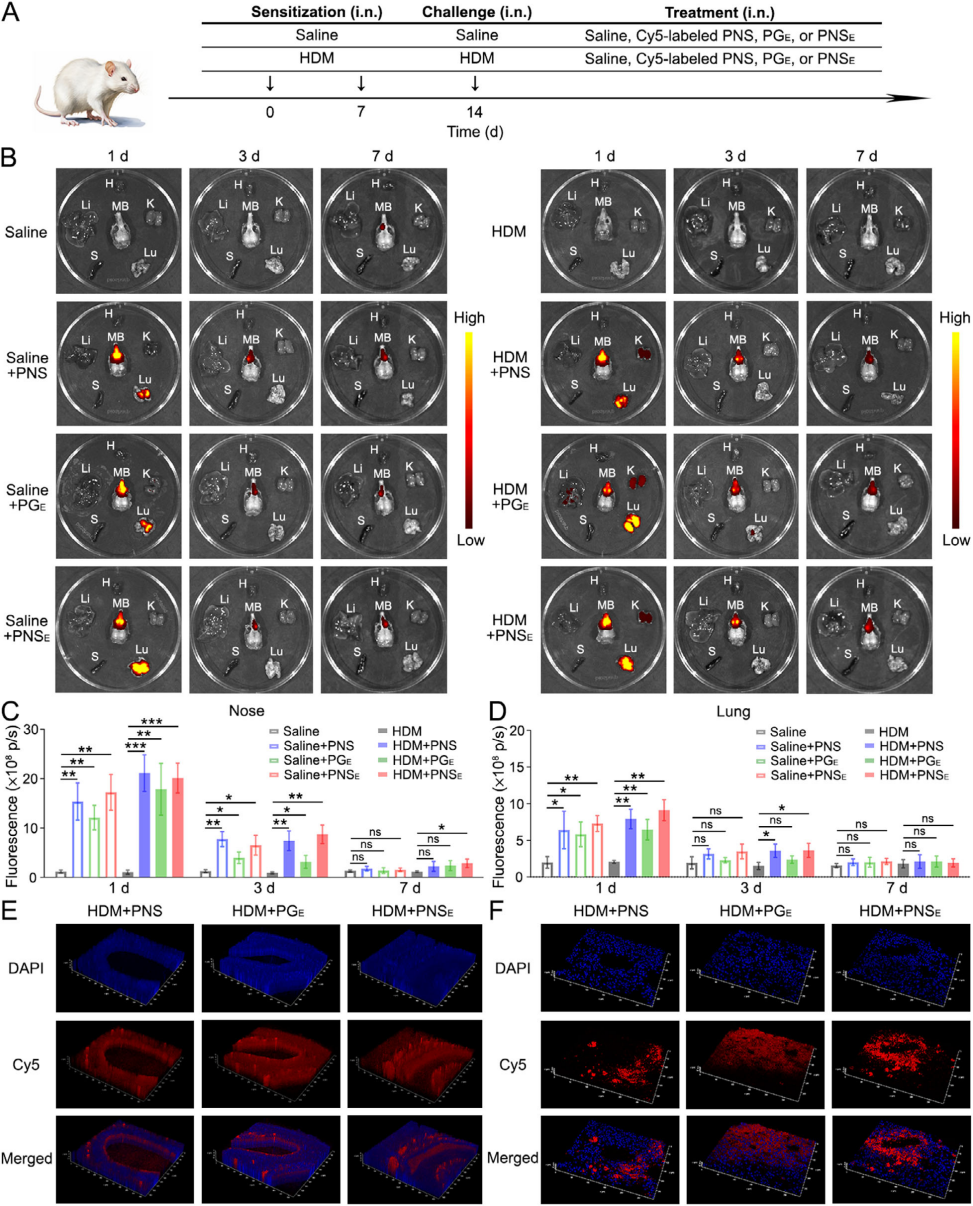
Biodistribution of organic nanosheets after intranasal instillation. A) schematic diagram outlining the experimental protocol. B) Fluorescence imaging of MBs and major organs of mice subjected to Saline, Saline+PNS, Saline+PG_E_, Saline+PNS_E_, HDM, HDM+PNS, HDM+PG_E_, and HDM+PNS_E_. PNS, PG_E_, and PNS_E_ were labeled by Cy5. H: Heart; Li: Liver; S: Spleen; Lu: Lung; K: Kidney; MB: Maxillary bones. Quantification of fluorescence intensity in C) noses and D) lungs of experimental mice on day 1 after treatment with Cy5‐labeled PNS, PG_E_, and PNS_E_. The 3D CLSM images of the slices of the E) nasal mucosa and F) lungs of experimental mice. Data represent mean ± S.D. (*n* = 3; two‐way ANOVA, ns represents not significant, *p < 0.05, **p < 0.01, ***p < 0.001).

Next, PNS, PG_E_, and PNS_E_ were labeled with Cy5 fluorescent dye to investigate their distribution after intranasal administration (**Figure** **5**A). Blood samples collected 24 hours post‐instillation showed no detectable fluorescent signals, indicating that minimal nanomaterials entered systemic circulation (Figure S21, Supporting Information). Major organs and MBs were also collected to analyze nanomaterial accumulation. Although the accumulation of PNS, PG_E_, and PNS_E_ was observed in MBs and lungs of all experimental mice on day 1, the fluorescent signals were substantially stronger in HDM‐treated mice compared to sham mice on day 3 and day 7 (Figure 5B–D). Mice with airway inflammation exhibit significantly greater nasal secretion volumes compared to healthy mice, mirroring clinical observations.^[^ ^1^, ^2^, ^3^, ^4^ ^]^ Consequently, the majority of nanosheets bound to epithelial sEVs were effectively cleared from the airways through this secretory mechanism. In contrast, the fluorescent signal strength in hearts, livers, spleens, and kidneys was nearly undetectable (Figure 5B; Figures S22,S23, Supporting Information), confirming the biosafety of organic nanosheets. 3D CLSM images of nasal and lung sections from treated mice on day 1 also revealed deeper penetration into nasal and lung tissues by PNS and PNS_E_ compared to PG_E_ (Figure 5E,F), proving airway inflammation‐targeting properties of PNS_E_, which is of great importance for future clinical therapy.

### 2.7 PNS_E_ Treatment Alleviated Upper‐Airway Inflammation

**Figure 6:**
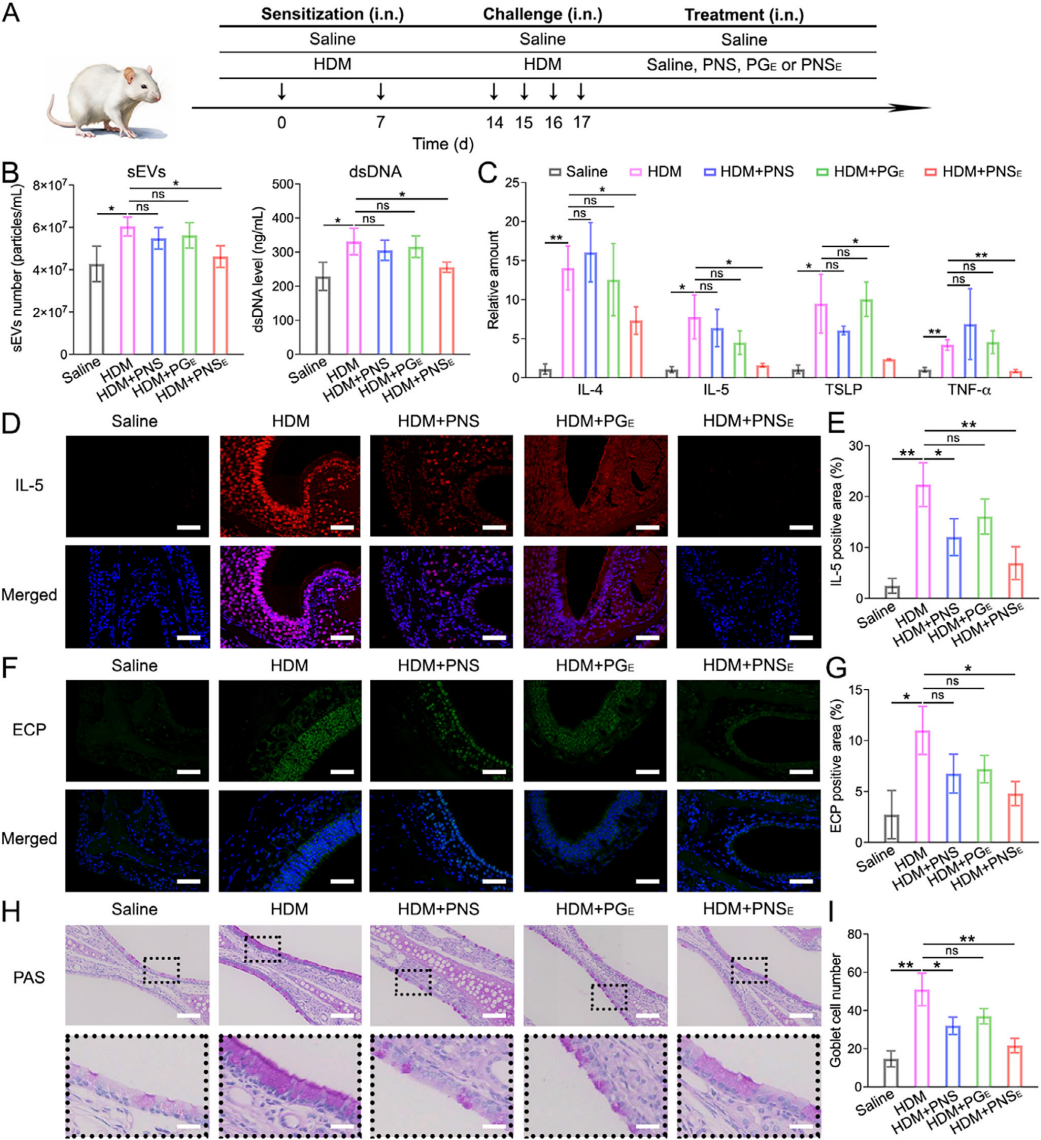
Modulation of upper airway inflammation by organic nanosheets. A) The experimental protocol of PNS_E_ treatment for model mice with allergic airway inflammation. B) The sEVs number and dsDNA level in BALF from experimental mice. C) The relative expression levels of IL‐4, IL‐5, TSLP, and TNF‐α in the nasal mucosa of experimental mice. D) Representative IL‐5 immunostaining images of the nasal mucosa (Scale bars: 50 µm) and lungs (Scale bars: 100 µm). E) Quantification of IL‐5 positive area in images. F) Representative ECP immunostaining images of the nasal mucosa (Scale bars: 50 µm) and lungs (Scale bars: 100 µm). G) Quantification of ECP‐positive area in images. H) Representative PAS staining images of the nasal mucosa (Scale bars: 100 µm; scale bars in amplified sections: 25 µm). I) Quantification of goblet cell number in images. Data represent the mean ± S.D. (*n *= 6, one‐way ANOVA; ns represents no significant difference, *p < 0.05, **p < 0.01).

To comprehensively evaluate the therapeutic effect of functional nanosheets on airway inflammation, BALF, MBs, and lungs from the experimental mice were collected (**Figure** **6**A).^[^ ^25^, ^38^ ^]^ Given the elevated sEV and dsDNA excretion by HDM‐stimulated epithelial, we assessed the sEV and dsDNA levels in the nasal lavage fluids (NALF) across various treatment groups. The results revealed the sEVs and dsDNA levels in HDM‐stimulated mice showed significant elevations (1.41‐fold and 1.45‐fold) compared to the Sham mice (Figure 6B). PNS_E_ treatment potently reduced sEV and dsDNA levels to 76.5% and 77.2%, respectively, and the reduction in the PNS and PG_E_‐treated mice is not significant. The expression and generation of pro‐inflammatory cytokines in the nasal mucosa and NALF were determined by qPCR and ELISA (Figure 6C; Figure S24, Supporting Information), and the levels of IL‐4, IL‐5, thymic stromal lymphopoietin (TSLP), and tumor necrosis factor‐α (TNF‐α) were found to be elevated in the HDM+Saline group. Additionally, the q‐PCR studies demonstrated that HDM+PNS_E_ treatment declined IL‐4, IL‐5, TSLP, and TNF‐α expression (25%, 42%, 21%, and 25%) in the nasal mucosa of inflammatory model mice (Figure 6C), which is also confirmed by ELISA results. As expected, the anti‐inflammation performance of PNS or PG_E_ was less efficient than PNS_E_, both in ELISA and PCR experiments.

In representative immunostaining images, increased IL‐5 and ECP expression was observed in the HDM group compared to the Saline group (Figure 6D–G). HDM+PNS_E_ treatment resulted in a decline in both the IL‐5 and ECP levels (reducing to ≈31% and 44%), suggesting an alleviation of allergic airway inflammation. However, the IL‐5 and ECP levels only decreased by 46% and 38% in the PNS group, and the reductions were insignificant in the PG_E_ group. In addition, periodic acid Schiff (PAS), eosinophil, and toluidine blue (TB) staining were exploited to evaluate goblet cell hyperplasia, eosinophil infiltration, and mast cell infiltration, respectively (Figure 6H,I; Figures S25,S26, Supporting Information).^[^ ^50^, ^51^ ^]^ Quantification data of the representative images revealed that PNS_E_ treatment potently reduced ≈58% of the goblet cell hyperplasia, 67% eosinophil infiltration, and 70% eosinophil infiltration in the nasal mucosa of model mice, respectively. Furthermore, PNS_E_ exhibited a more robust anti‐inflammation effect compared to PNS and PG_E_ in most experiments. Collectively, these results suggested that the introduction of Apt_E_ onto organic nanosheets to create functional 2D nanoplatforms effectively ameliorated allergic inflammation in the upper airway.

#### 2.7.1 PNS_E_ Treatment Alleviated Lower‐Airway Inflammation

**Figure 7:**
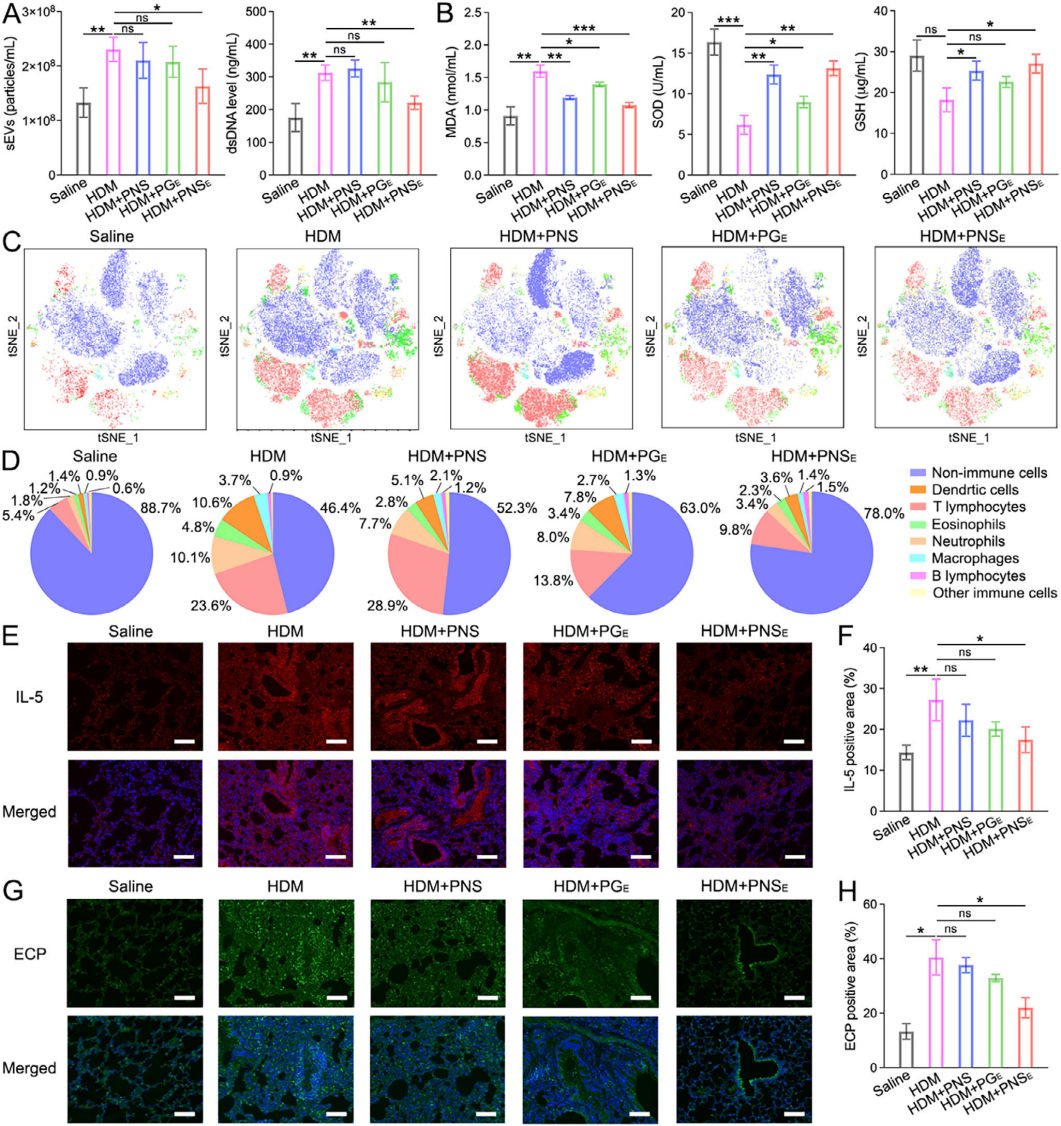
Modulation of lower airway inflammation by organic nanosheets. A) The sEVs number and dsDNA level in BALF from experimental mice. B) MDA, SOD, and GSH concentration in BALF. C) t‐SNE analysis based on the flow cytometry results of lung cells from experimental mice. D) The ratio of different cell types in the lungs calculated by flow cytometry. E) Representative IL‐5 immunostaining images of the lungs (Scale bars: 100 µm). F) Quantification of IL‐5 positive area in images. G) Representative ECP immunostaining images of the lungs (Scale bars: 100 µm). H) Quantification of ECP‐positive area in images. Data represent the mean ± S.D. (*n* = 6, one‐way ANOVA; ns represents no significant difference, *p < 0.05, **p < 0.01, ***p < 0.001).

It was broadly recognized that allergic upper‐airway inflammation was closely related to the lower‐airway inflammatory disorder, a concept known as “one airway, one disease”.^[^ ^2^, ^3^, ^5^ ^]^ Intranasal HDM instillation caused allergic inflammation in the entire airway of model mice.^[^ ^13^ ^]^ Therefore, the anti‐inflammation therapeutic effects of PNS_E_ for the lungs were also carefully evaluated. Given the elevated sEV and dsDNA excretion by HDM‐stimulated epithelial, we assessed the sEV and dsDNA levels in the BALF across various treatment groups (**Figure** **7**A). Compared to the sham (Saline group), mice treated with HDM followed by saline (HDM+Saline group) showed a significant increase in sEV (1.73‐fold) and dsDNA (1.78‐fold) levels. Treatment with PNS_E_ markedly reduced sEV levels and dsDNA concentration by 21.6% and 22.1%, respectively, and the reduction was obviously higher than that in the PNS and PG_E_ groups. In addition, oxidative stress was determined by the concentrations of glutathione (GSH), superoxide dismutase (SOD), and malondialdehyde (MAD) in the BALF (Figure 7B).^[^ ^43^, ^44^, ^52^ ^]^ PNS_E_ treatment significantly increased GSH and SOD levels (1.49‐fold and 2.13‐fold, respectively) and decreased MDA levels (to 67.4%) compared to the HDM‐induced inflammatory group. Additionally, BLAF from the experimental mice was collected, and the levels of IL‐4, IL‐5, TSLP, and TNF‐α declined in the inflammatory mice after PNS_E_ treatment, confirming the anti‐inflammatory effect (Figure S27, Supporting Information).

Next, the lungs isolated from experimental mice were digested to a single‐cell suspension and subjected to flow cytometry. The multi‐channel results of flow cytometry were analyzed by t‐distributed stochastic neighbor embedding (t‐SNE), and immune cell populations, including DCs, T lymphocytes, eosinophils, neutrophils, macrophages, and B lymphocytes, were clustered (Figure 7C).^[^ ^53^, ^54^ ^]^ HDM instillation significantly increased the infiltration of immune cells, with total immune cells rising from 11.3 ± 7.6% to 53.6 ± 5.7%, especially DCs (7.6‐fold increase) and T lymphocytes (4.4‐fold increase) (Figure 7D; Figure S28, Supporting Information). Consistent with the anti‐inflammation effect in the nasal mucosa, the percentage of total inflammatory cells descended to 22 ± 5.4% after PNS_E_ treatment; among them, the percentage of DCs and T lymphocytes was reduced by 66.2% and 58.4%, respectively. Further analysis revealed PNS and PG_E_ treatments only declined the percentage of inflammatory cells to 47.7 ± 5.9% and 37 ± 7.2%, which are much lower than those of the PNS_E_ groups. The results indicated that PNS_E_ facilitated a more balanced immune cell distribution compared to PNS and PG_E_ groups and was more similar to the sham group.

Histological analysis, including H&E, eosinophil, PAS, IL‐5, ECP, cytokeratin‐5 (CK‐5), and cytokeratin‐13 (CK‐13) staining, was conducted on lung tissues from experimental mice.^[^ ^38^, ^55^ ^]^ Quantification data revealed that the IL‐5 and ECP‐positive area in lung slices from HDM‐stimulation mice was statistically higher than that in these saline‐instilled mice, confirming the provoking of allergic airway inflammation (Figure 7E–H). Due to the robust anti‐inflammation ability, the IL‐5 and ECP levels were reduced by 35.8% and 45.7% in PNS_E_‐treated mice, respectively. In addition, H&E, eosinophil, and PAS staining demonstrated severe immune cell infiltration and goblet cell hyperplasia in the bronchus of mice with allergic inflammation (Figure S29–S31, Supporting Information). Consistently, PNS_E_ treatment alleviated these pathological changes in the inflammatory area, which is attributed to the elimination of epithelial sEVs and reactive species. However, the aforementioned therapeutic effects were insignificant or less effective in PNS and PG_E_ groups, proving the importance of rational design in anti‐inflammation nanomedicine construction. Further, a parallel study of immunostaining for CK‐5 and CK‐13, a marker of epithelial tissues, was performed on the lungs of the experimental mice to evaluate epithelial tissue integrity and health. Allergen contact and sensitization damaged airway epithelial cells, proved by a decrease of CK‐5 and CK‐13 staining observed in the HDM, HDM+PNS, and HDM+PG_E_ groups (Figures S32,S33, Supporting Information). Interestingly, the injured epithelial was attenuated after PNS_E_ administration, meaning the modulation of uncontrolled inflammatory response is beneficial for epithelial repair.

### 2.8 Transcriptome Analysis

**Figure 8:**
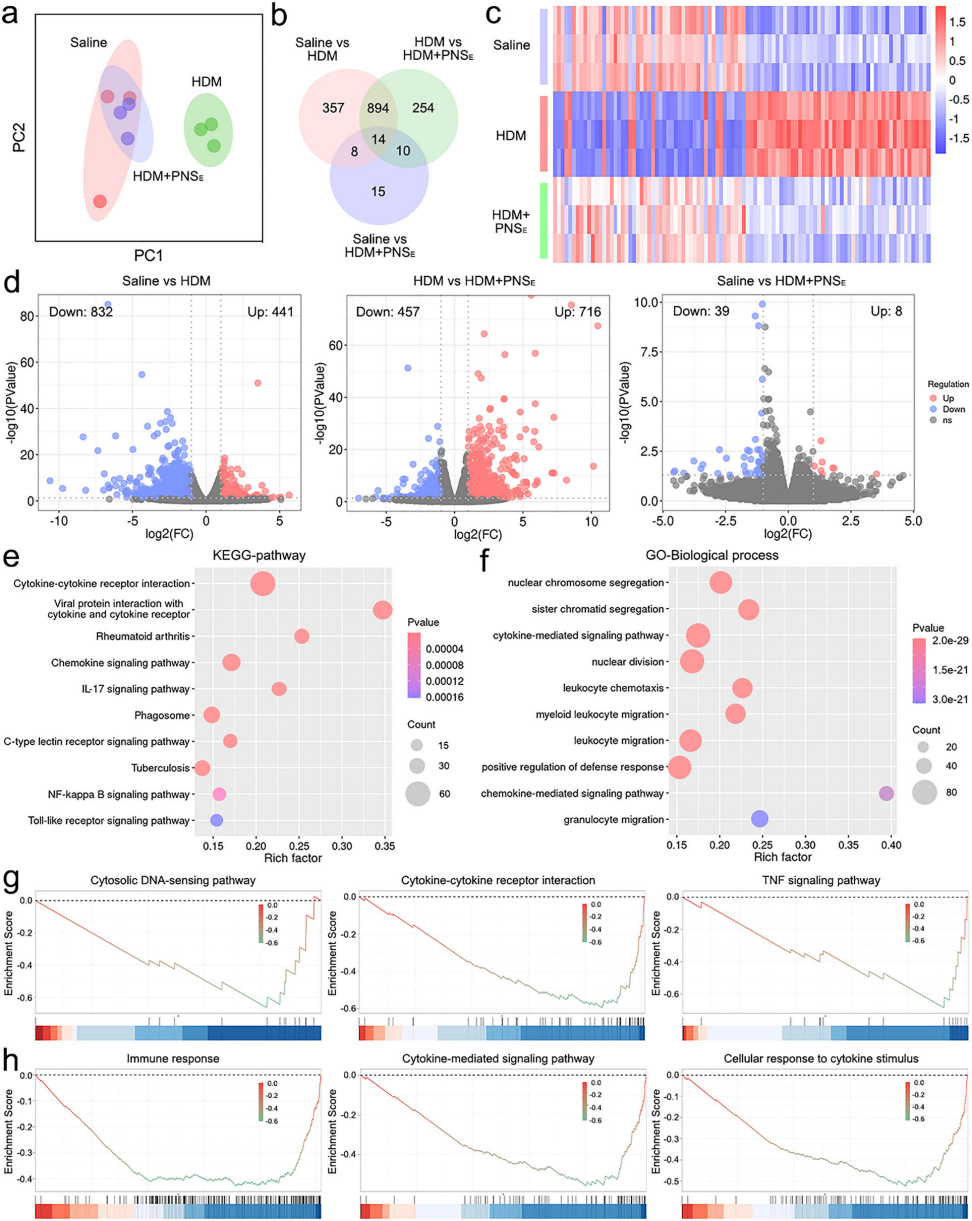
Transcriptome analysis based on RNA‐seq results. A) PCA results of the Saline group, HDM group, and HDM+PNS_E_ group. B) Venn map of DEGs between the experimental groups. C) Heat map of the first 100 DEGs with significant changes between the Saline group, HDM group, and HDM+PNS_E_ group. D) Volcano maps based on the Saline versus HDM group, HDM versus HDM+PG‐NS_E_ group, and Saline versus HDM+PNS_E_ group. E) KEGG pathway analyses of DEGs between the HDM group and the HDM+PG‐NS_E_ group. F) GO term (biological process) enrichment analyses of DEGs between the HDM group and HDM+PNS_E_ group. G) GSEA results based on the KEGG gene set (HDM vs HDM+PNS_E_). The dotted line represents the zero line. H) GSEA results based on the GO analysis (HDM vs HDM+PNS_E_). The dotted line represents the zero line.

On the basis of the above‐mentioned analysis of anti‐inflammation therapeutic results, RNA sequencing and transcriptome analysis on lung tissues were performed to evaluate the therapeutic effects of PNS_E_ on allergic airway inflammation.^[^ ^56^ ^]^ Distinct transcriptomic differences between the HDM and saline groups were observed in principal component analysis (PCA) of the generated gene expression matrix, while the mice treated with HDM+PNS_E_ displayed a closer correlation to the sham group (**Figure** **8**A). The transcriptomic analysis revealed 1273 and 1172 differentially expressed genes (DEGs) in the Saline versus HDM and HDM versus HDM+PNS_E_ comparisons (908 overlapping genes), respectively (Figure 8B; Figure S34, Supporting Information). In contrast, much less difference (47 DEGs) was identified in the Saline versus HDM+PNS_E_ comparison, indicating the treatment showed the ability to reverse the allergic pathological conditions and restore a healthy status. Subsequently, the expression levels of the top 100 DEGs were summarized, and the outcomes confirmed the perturbations of gene expression in inflammatory tissues were largely restored after PNS_E_ treatment (Figure 8C). To further distinguish the upper‐regulated or down‐regulated genes, volcano plots were employed (log_2_|FC| > 1, *p* < 0.05), and 441 upregulated and 832 downregulated genes were found in the HDM group compared to the saline group (Figure 8D). In contrast, the results for the HDM versus HDM+PNS_E_ were 716 upregulated and 457 downregulated genes, respectively, while only 8 upregulated and 39 downregulated genes were found in the saline versus HDM+PNS_E_ comparison. Additionally, the analysis by Minus‐versus‐Add (MA) plots also showed the number of DEGs in Saline versus HDM+PNS_E_ was much lower than in the other two comparisons (Figure S35, Supporting Information). These results jointly confirmed that PNS_E_ treatment effectively restored gene expression levels of mice with allergic inflammation toward the saline baseline.

Kyoto encyclopedia of genes and genomes (KEGG) analysis was performed to analyze the enriched pathways of DEGs in Saline versus HMD and HDM versus HDM+PNS_E_ groups.^[^ ^57^ ^]^ The DEGs were mainly involved in cytokine–cytokine receptor interaction, IL‐17 signaling pathway, NF‐κB signaling pathway, chemokine signaling pathway, etc. (Figure 8E; Figures S36,S37, Supporting Information). On the other hand, gene ontology (GO) analysis for biological processes demonstrated that the enriched pathways were predominantly associated with cytokine‐mediated signaling pathways, leukocyte chemotaxis, granulocyte migration, inflammatory response, etc. (Figure 8F; Figures S38,S39, Supporting Information).^[^ ^58^ ^]^ Further gene set enrichment analysis (GSEA) analysis of KEGG‐enriched pathways showed that the cytosolic DNA‐sensing pathway, cytokine–cytokine receptor interaction, and TNF signaling pathway were down‐regulated in HMD versus HDM+PNS_E_ comparison (Figure 8G). In correspondence to KEGG outcomes, GSEA analysis of GO biological processes also indicated a down‐regulation of several critical immune responses and inflammatory pathways after PNS_E_ treatment, comprising immune response, cytokine‐mediated signaling pathway, and cellular response to cytokine stimulus (Figure 8H). In summary, these results revealed that PNS_E_ effectively reversed HDM‐induced allergic airway inflammation, indicating the potential as a novel therapeutic intervention for AAD patients.

## 3 Discussion

Allergic airway inflammatory diseases, including AR and asthma, have a serious suboptimal impact on patients' quality of life and impose a substantial economic burden on healthcare systems.^[^ ^1^, ^2^, ^3^, ^4^ ^]^ Worryingly, ≈40% of AR patients eventually progress to asthma, a chronic and refractory lower‐airway disorder that can become life‐threatening.^[^ ^5^ ^]^ Current clinical treatments for AAD primarily rely on glucocorticoids, while the therapeutic efficacy remains unsatisfactory. As a result, the rising prevalence of AAD and the limitations of existing treatments underscore the urgent demand for innovative approaches to better manage and treat these conditions. DCs play a governing role in allergic airway inflammation, especially the antigen‐presenting capacity, which is closely associated with allergic inflammatory response.^[^ ^7^, ^8^, ^9^ ^]^ In addition to DCs, eosinophils were also reported to be heavily involved in the inflammatory response of AAD patients.^[^ ^7^, ^8^, ^9^ ^]^ Under sensitization, eosinophils degranulate and form EETs through a process called EETosis, which contributes to chronic eosinophilic inflammation. Consequently, regulating DC overactivation and EET formation may represent new therapeutic strategies for managing allergic inflammation.

Previous reports manifested that sEVs and the incorporated dsDNA were tightly associated with various inflammatory diseases, including airway disorders, Crohn's disease, and dermatomyositis.^[^ ^12^ ^]^ It is well‐established that dsDNA is released from the cells of damaged tissues following infection or physical injury. For example, dsDNA release from rhinovirus (RV)‐infected nasal mucosa cells has been observed, whereby dsDNA levels were strongly related to symptom severity. In this study, we found that sEVs and dsDNA levels were increased in the nasal secretions of AR patients versus those of healthy individuals (Figure 2A–C), as well as in the BALF of model mice with allergic airway inflammation versus that of sham mice. Moreover, we observed positive correlations between sEVs number, dsDNA concentration, and allergic inflammatory cytokines (IL‐4 and IL‐5) level in the nasal secretions of AR patients (Figure 2D). These findings implied the potential involvement of sEVs and dsDNA in the pathogenesis of allergic inflammation and highlighted their association with DC maturation and EET formation. To probe the underlying mechanisms, BMDCs and eosinophils were exposed to allergen‐stimulated epithelial sEVs. Our results disclosed that the above sEVs elicited DC activation and EET formation, which could be suppressed by blocking the STING pathway, confirming the crucial role of dsDNA within sEVs in driving the allergic inflammatory response (Figure 2E,F). To further examine whether sEVs could act as a potential therapeutic target in AAD treatment, model mice with HDM‐triggered allergic airway inflammation were established.^[^ ^30^, ^31^, ^35^ ^]^ GW4869, a neutral sphingomyelinase inhibitor, was administrated to reduce the sEVs generation^[^ ^16^, ^36^ ^]^ in the inflamed airway tissues of mice, which led to decreased IL‐5 level and EET formation both in upper‐airway and lower‐airway tissues (Figure 2G–J). The experimental results revealed pretreatment with GW4869 attenuated the allergic inflammation in the HDM‐stimulated model mice. Nevertheless, its clinical application is markedly restricted by suboptimal safety profiles and associated side effects. Therefore, there is a pressing need to investigate other biocompatible sEVs removal strategies for the clinical treatment of AAD patients.

In our previous studies, functionalized MoS_2_ nanosheets were developed to capture tumor‐derived sEVs, successfully inhibiting cancer progression and metastasis.^[^ ^19^ ^]^ The advantages of a 2D nanostructure in capturing sEVs, such as planar geometry, flexible backbone, and multivalent interactions, were demonstrated. However, the clinical use of inorganic nanosheets like graphene, MoS_2_, TiS_2_, and MXene is limited due to incomplete degradation, accumulation, and toxicity.^[^ ^19^, ^20^, ^21^, ^22^ ^]^ To address this limitation, we developed “inorganic‐free” PG‐based nanosheets (PNS) by degrading the TiS_2_ backbone. PG was selected for its ease of preparation, functionalization, and excellent biocompatibility.^[^ ^23^, ^24^, ^25^ ^]^ Its protein‐resistant properties, stemming from improved interactions with water molecules, also enhance biocompatibility. Given that aptamers are oligonucleotides with high specificity and affinity for the protein targets and EGFR is highly expressed on epithelial sEVs,^[^ ^26^ ^]^ we conjugated Apt_E_ to the organic nanosheets (PNS_E_) to enhance sEV binding.

The successful preparation of “inorganic‐free” nanosheets was verified by planar geometry in AFM images and the disappearance of the Ti peak in XPS results (Figure 3A,B). The cell experiments revealed that PG, PNS, PG_E_, and PNS_E_ exhibited similarly low cytotoxicity to that of TP, confirming the improvement of biocompatibility after the degradation (Figure 4C). Moreover, we found that the PNS_E_ exhibited a higher sEVs binding capacity than the PNS and PG_E_ (Figure 4D,E), highlighting the critical role of the 2D nanostructure and targeting aptamers in the construction of sEVs scavengers. In addition to allergen‐stimulated sEVs, excessive reactive species are known to contribute to persistent allergic inflammation.^[^ ^43^, ^44^ ^]^ The organic nanosheets, composed of PG and thiol (─SH) groups, exhibited robust RONS reduction capabilities, converting them into more stable and less reactive molecules (Figure 4H,I).

We hypothesized that aptamer‐functionalized organic nanosheets could bind epithelial sEVs to form a stable complex, thereby inhibiting DC maturation and EET formation. Cellular uptake and intracellular colocalization of epithelial sEVs were assessed after incubation with PNS, PG_E_, and PNS_E_. Although flow cytometry displayed that the uptake efficacy of sEVs was similar in different groups, more intracellular colocalization with PNS_E_ was observed in CLSM images compared to the other two groups (Figure 4A,B). The potent binding of sEVs with PNS_E_ in intricate intracellular conditions is promising to suppress the STING activation and the following allergic inflammation. Previous studies have already shown that HDM incubation had the ability to induce the release of sEVs from injured airway epithelial cells, which induced DC maturation via STING activation. Further, eosinophils primarily recognize allergen‐stimulated epithelial sEVs, which subsequently trigger EETosis, a multistep process through which eosinophils release EETs.^[^ ^25^, ^38^ ^]^ The DC activation and EET production have been closely associated with the progression of allergic inflammation via this cascade of events. Therefore, we further examined whether the developed sEVs scavenging strategy could suppress dysregulated activation of DC and overmuch formation of EETs. Consistent with the outcomes of previous sEVs binding studies, the PNS_E_ nanosheets reduced the percent of CD11c^+^/CD80^+^/CD86^+^, CD11c^+^/CD40^+^, and CD11c^+^/MHC II^+^ cells in BMDCs, which could result in alleviated allergic inflammatory response (Figure 4C,D). In addition, PNS_E_ also inhibited the degranulation of eosinophils and reduced the ECP‐positive area after treatment (Figure 4E,F). The PNS_E_ possesses a larger surface area than the same volume of PG_E_, providing a greater number of exposed edges and faces for sEVs binding. These findings highlight the potent sEV elimination properties of our functional organic nanosheets and their potential application in treating conditions associated with allergen sensitization.

Building on this discovery, the functional nanosheets were comprehensively evaluated to assess their potential biosafety and biocompatibility in vivo using the model mice. The results underscore the favorable biosafety profile of the functional organic nanosheets as a safe and biocompatible nanoplatform for future clinical translation. We conducted additional investigations to evaluate the capacity of functional organic nanosheets to exclusively mop up sEVs and modulate allergic airway inflammation. Although the therapeutic effects are unsatisfactory, topical application of corticosteroids, antihistamines, and membrane stabilizers has been widely exploited in the clinic for treating AAD.^[^ ^6^, ^13^, ^14^ ^]^ Therefore, after careful consideration, we determined that intranasal (i.n.) instillation would be the ideal route for the nanomedicine administration for model mice with airway disorders. Building on this foundation, we developed an effective noninvasive delivery system for anti‐inflammatory nanomedicines to treat allergic airway inflammation.

The biodistribution study revealed that PNS and PNS_E_ preferentially accumulated in inflamed airway tissues, with higher fluorescence in the noses and lungs of model mice compared to sham mice (Figure 5A–D). This accumulation, coupled with the lack of detectable nanosheets in blood or major organs, explains the superior biocompatibility of PNS_E_. The nanosheets exhibited deep tissue infiltration and prolonged retention at inflamed sites, leading to enhanced permeation and retention in inflamed tissues (Figure 5E,F). These findings highlight the targeted and selective accumulation capabilities of PNS_E_, which are crucial for AAD treatment.

In terms of in vivo sEVs scavenging ability, our study demonstrated that PNS_E_ nanosheets reduced the sEVs and dsDNA levels in NALF and BALF of mice with allergic airway inflammation, which is more effective than PNS and PG_E_ (Figure 6B,7A). Oxidative stress during inflammation can deplete GSH levels, impairing cellular function and causing increased damage.^[^ ^43^, ^44^, ^52^ ^]^ Additionally, SOD and MDA, a byproduct of lipid peroxidation, are commonly known oxidative stress markers. The in vivo antioxidant capacities of organic nanosheets were also confirmed in BALF (Figure 7B). Notably, the cytokine levels, including IL‐4, IL‐5, TSLP, and TNF‐α in NALF and BALF, which can exacerbate allergic inflammation, were reduced after PNS_E_ treatment. The therapeutic efficacy outperforms PNS and PG_E_‐treated mice, and the results were also confirmed by qRT‐PCR analysis and immunostaining analysis.

Inflammatory cell infiltration, including eosinophils and mast cells, leads to dysregulated inflammation and elevated Th2 cytokines.^[^ ^7^, ^8^, ^9^ ^]^ Flow cytometry with t‐SNE analysis^[^ ^53^, ^54^ ^]^ showed significant immune cell infiltration in HDM‐treated mice compared to sham mice, which was mitigated by PNS_E_ (Figure 7C,D). Immunostaining further demonstrated reduced eosinophils, EETs, and mast cell degranulation in PNS_E_‐treated mice (Figure 6, 7). PNS_E_ also significantly reduced goblet cell hyperplasia and preserved airway epithelial integrity. These results emphasize the anti‐inflammatory efficacy of PNS_E_, positioning it as a promising tool for anti‐inflammatory nanomedicine.

Transcriptome analysis was exploited in the lungs of model mice to obtain a more comprehensive analysis of the therapeutic effect of PNS_E_ on allergic airway inflammation (Figure 8).^[^ ^56^, ^57^, ^58^ ^]^ The results revealed that PNS_E_ treatment modulated the immune response, inflammatory pathways, chemokine signaling, cytokine interaction, leukocyte/granulocyte migration, and cytosolic DNA‐sensing pathways. For instance, the cytosolic DNA‐sensing pathways, including cGAS‐STING and toll‐like receptors, have emerged as key mediators in inflammation, tumors, and autoimmune diseases.^[^ ^59^, ^60^ ^]^ Among them, the cGAS‐STING signaling pathway mediates interferon when the cGAS receptor can bind to dsDNA, activating STING and then inducing downstream signals, such as IFN regulatory factor 3 (IRF3). It was reported that the cGAS‐STING signaling pathway was closely associated with the allergic inflammatory cascades, involving DC maturation and granulocyte degranulation. On the other hand, the dysregulation of the airway immune system through the NF‐κB signaling pathway can lead to airway oxidative stress and injury.^[^ ^61^ ^]^ NF‐κB regulates the balance of Th1/Th17 by affecting the maturation and migration of DCs, which in turn affects the development of allergic inflammation, and blocking the NF‐κB signaling pathway is a promising therapeutic target for AAD. In addition, it was found that Th17 cells and IL‐17 promoted eosinophil and neutrophil expression in AR and asthma patients, indicating the dominant role in allergic inflammatory response.^[^ ^62^ ^]^ The elevated levels of IL‐17 mRNA, as well as IL‐17 protein, have been found in the lungs, sputum, tracheal aspirate, bronchoalveolar lavage, and sera from asthmatic patients, and IL‐17 was also related to resistance to steroids. In our study, all the aforementioned allergic inflammatory pathways were suppressed after PNS_E_ treatment, supporting the therapeutic effects of functional organic nanosheets on alleviating allergic airway inflammation.

## 4 Conclusion

In summary, epithelial sEVs and the incorporated dsDNA were found to be elevated in the nasal secretions of AR patients and were positively correlated with allergic inflammation. It was then revealed the DC maturation and EET formation of allergic inflammation were associated with the STING pathway stimulated by dsDNA in EVs. In addition, we showed that suppression of sEV generation by GW4869 mitigated allergic inflammation in a mouse model. To avoid biological toxicities and boost the prospect of clinical application, an alternative sEVs removal strategy by functional nanosheets was developed. In this approach, “inorganic‐free” nanosheets were fabricated by degrading the inorganic backbone, and Apt_E_ was modified to promote the affinity to epithelial sEVs. The functionalized nanosheets PNS_E_ suppressed sEVs‐induced STING activation and EET formation. Further, organic nanosheets also displayed robust anti‐oxidant capacity, which is also significant for the treatment of allergic inflammation. Notably, PNS_E_ preferentially accumulated in the inflamed airway tissues, where it modulated DC maturation, EET formation, goblet cell hyperplasia, mast cell infiltration, and reduced the level of allergic inflammatory cytokines. Moreover, flow cytometry with t‐SNE analysis and transcriptome analysis with RNA‐seq of airway tissues further proved the alleviation of allergic inflammation in animal models. Our work presents a novel sEV elimination strategy using functional organic nanosheets, offering an effective intervention for allergic airway inflammation and providing new insights into epithelial sEV‐related inflammatory pathways. In addition, this study also highlights the importance of the 2D nanostructure and aptamer modifications in the design of clinical translational sEVs scavengers.

## 5 Experimental Section

### Ethics Statements

Twenty AR patients and 20 healthy volunteers in the Sixth Affiliated Hospital and First Affiliated Hospital of Sun Yat‐sen University were included in this study. Nasal secretions were collected with an expansive sponge from these patients, which was approved by the ethics committee of Sun Yat‐sen University (2023ZSLYEC‐497). All patients were informed of the purpose of the donated clinical samples and signed informed consent forms.

Animal experiments were approved by the ethics committee of the Sixth Affiliated Hospital, Sun Yat‐sen University (No. IACUC‐2023021601, SLBH‐202411200002). Adult female BALB/c mice (4 to 6 weeks, 20 ± 2 g) were provided by YaoKang company (Guangdong) and were raised in a specific pathogen‐free environment at Sixth Affiliated Hospital, Sun Yat‐sen University.

### Statistical Analysis

All statistical analyses were conducted using GraphPad Prism 10. Prior to applying parametric statistical tests, the normality of data distributions was assessed using the Shapiro–Wilk test. Differences between two independent groups with one nominal‐level independent variable were evaluated using an unpaired two‐tailed Student's *t‐*test. For comparisons involving three or more groups with a single independent variable, one‐way analysis of variance (ANOVA) was conducted. For designs with two independent variables, two‐way ANOVA was performed, with post‐hoc tests selected based on the experimental design. All post‐hoc *p*‐values reported in figures were adjusted for multiple comparisons using the specific method (Tukey's or Dunnett's) recommended by Prism for each analysis. Data were displayed as mean ± S.D., and sample size (*n*) for each statistical analysis was indicated in the experiment section and figure legends. Asterisks represent the following *p*‐values: *p < 0.05, **p < 0.01, and ***p < 0.001 in displayed figures. Unless otherwise stated, each mean and S.D. was derived from a minimum of three experimental units or subjects.

## Conflict of Interest

The authors declare no conflict of interest.

## Author Contributions

Conceptualization: Z. Tu, Y. Wen, J. Li, and W. Wen, Methodology: Z. Tu, J. Lin, C. Xu, Z. Li, Y. Zhu, Z. Qiu, and Q. Wang, Investigation: Z. Tu, C. Xu, Z. Li, Y. Zhu, and S. Zhang, Visualization: Z. Tu, J. Lin, C. Xu, and Z. Li, Supervision: Z. Tu, K. W. Leong, Y. Wen, J. Li, and W. Wen, Writing—original draft: Z. Tu and Y. Zhu, Writing—review & editing: Z. Tu, Y. Wen, J. Li, and W. Wen

## Supporting information



Supporting Information

## Data Availability

All data supporting the findings of this research are available within the article and its supplementary information or from the corresponding author upon reasonable request.
