# Supplementary material for: Tackling Allergic Airway Inflammation with Organic Sheet‐Like Nanoplatforms by Targeted Elimination of Epithelial Small Extracellular Vesicles
Source: Adv Sci (Weinh). 2025 Sep 26;12(46):e04197. doi: 10.1002/advs.202504197 (PMC12697784; doi:10.1002/advs.202504197)
Supplement: Supplementary file 1 — Supporting Information [file ADVS-12-e04197-s001.pdf]

Supplementary Materials

**Tackling Allergic Inflammation with Organic Sheet-like Nanoplatforms by Targeted Elimination of Epithelial Small Extracellular Vesicles**

Zhaoxu Tu*, Junyan Lin, Zhixin Li, Yuefei Zhu, Changyi Xu, Zihan Qiu, Qiumin Wang, Yang Ye, Yihui Wen*, Jian Li*, Kam W. Leong, and Weiping Wen*

Z. Tu, J. Lin, Z. Li, C. Xu, Z. Qiu, Y. Ye, W. Wen
Department of Otolaryngology, The Sixth Affiliated Hospital, Sun Yat-sen University, Guangzhou 510655, Guangdong, China

Q. Wang, Y. Wen, J. Li, W. Wen
Department of Otolaryngology, The First Affiliated Hospital, Sun Yat-sen University, Guangzhou 510080, Guangdong, China

Y. Zhu, K. W. Leong
Department of Biomedical Engineering, Columbia University, New York 10027, NY, USA

Z. Tu, J. Lin, Z. Li, C. Xu, Z. Qiu, Y. Ye, W. Wen
Biomedical Innovation Center, The Sixth Affiliated Hospital, Sun Yat-sen University, Guangzhou 510655, Guangdong, China

Z. Tu, J. Lin, Z. Li, C. Xu, Z. Qiu, Y. Ye, W. Wen
Key Laboratory of Human Microbiome and Chronic Diseases (Sun Yat-sen University), Ministry of Education, China

## Materials and Methods

*Materials*

Titanium sulfide ($TiS_2$) powder, n-butyllithium solution (2.0 M in cyclohexane), lipoic acid (LA), glutamate (Glu), Horseradish Peroxidase (HRP), bovine serum albumin (BSA), 1-ethyl-3-(3-dimethyllaminopropyl)carbodiimide hydrochloride (EDC.HCl), and *N-hydroxysuccinimide* (NHS) were purchased from Sigma-Aldrich. Methanol, ethanol, N,N-dimethylformamide (DMF) and tetrahydrofuran (THF), N-methyl pyrrolidone (NMP), *dimethyl* sulfoxide (DMSO), n-hexane, and paraformaldehyde (4% PFA) were provided by Millipore-Sigma. 4',6-diamidino-2-phenylindole (DAPI), picogreen DNA assay, Cell Counting Kit-8 (CCK8) assay, exosome-depleted serum, QUANTI-Blue™ for alkaline phosphatase (ALP) detection, dichlorodihydrofluorescein diacetate (DCFH-DA), and TRIzol reagent were bought from Fisher Scientific.

EGFR aptamer (5' to 3': HOOC-TAC CAG TGC GAT GCT CAG TGC CGT TTC TTC TCT TTC GCT TTT TTT GCT TTT GAG CAT GCT GAC GCA TTC GGT TGA C) was synthesized by Sangon Biotech (Shanghai) Co., Ltd. GW4869 was bought from MedChemExpress (MCE). HS-PEG-NHS (Mw=1000) was bought from Ruixibio Biological Technology Co., Ltd. Glutathione (GSH), malondialdehyde (MDA), and superoxide dismutase (SOD) detection kits were purchased by Solarbio (Beijing, CHN). Human and murine ELISA kits of IL-1β, IL-6, and TNF-α were purchased from Invitrogen (US). Fluorescein isothiocyanate isomer I (FITC), PKH67, and Cyanine 5 (Cy5)-NHS ester were obtained from Lumiprobe Corporation (FL, USA). iScript cDNA synthesis kit and iTaq Universal SYBR Green Supermix were purchased from Bio-Rad. Milli-Q water was used in all experiments.

*Equipment*

Zeta potential and dynamic light scattering (DLS): NanoBrook 90Plus PALS (Brookhaven, US).

Fourier transform infrared spectroscopy (FTIR): Jasco FT/IR-4100 spectrometer. Transmission electron microscopy (TEM): FEI Tecnai G2 F30 TEM. Uranyl acetate negative staining was employed to record TEM images of sEVs. Atomic force microscopy (AFM): MultiMode Nanoscope V scanning probe microscopy (SPM) system (Bruker, USA). X-ray photoelectron spectroscopy (XPS): Nexsa (Themor Fisher) (10-400 μm). The data were processed using the UNIFIT program. Nanoparticle tracking analysis (NTA): NanoSight NS300 (Malvern, UK). Fluorescence: JASCOFP-6500 Spectrofluorometer. Quanti-Blue and CCK8 assays: microplate reader (Varioskan Flash, Thermo Fisher). Flow cytometry: Beckman Coulter flow cytometer (CytoFLEX S, Beckman Coulter). Quantitative polymerase chain reaction (qPCR): Applied Biosystems® QuantStudio™ 7 Flex (Thermo Fisher). Confocal laser scanning microscopy (CLSM): Leica TCS SP8 inverted microscope with an A1 scanning confocal unit. Image J software was employed to calculate the specified color area in CLSM images. H&E, PAS staining slices scanning: SQSL-510 automated slice scanning system (Shenzhen Shengqiang Technology Co, Ltd.). Biodistribution fluorescent images: IVIS Spectrum system (PerkinElmer, USA).

*Clinical sample analysis*

The nasal secretions were centrifuged at 100 g (10 min), 2,000 g (10 min), and 10,000 g (30 min) to remove intact cells, dead cells, and cell debris, respectively. Finally, sEVs were collected in the pellets after ultracentrifuged at 100,000 g (70 min). The number, size, and morphology of sEVs were measured by NTA, DLS, and TEM. The protein composition in sEVs was analyzed by WB, and the dsDNA concentration inside was determined by a pico-green assay.

*sEVs-induced STING activation*

RPMI2650 cells ($1.8 \times 10^6$ cells/dish) were incubated with HDM (1 μg/mL and 5 μg/mL) for two days. After that, the culture medium was replaced with DMEM containing 10% FBS (deactivated exosome-depleted) and then incubated for another two days. Finally, the culture medium was collected and centrifugated at 300 g (10 min) to remove intact cells, 2,000 g (10 min) to remove dead cells, and 10,000 g (30 min) to remove cell debris. Supernatants were collected and centrifuged at 100,000 g (70 min) to obtain sEVs(H) in pellets. During the same time, sEVs(N) were isolated from the cells incubated in a standard medium without HDM. The sEVs were washed in PBS, and protein concentration was determined using a Pierce BCA Protein Assay Kit (Thermo Fisher Scientific, USA).

HEK-blue™ STING (HEK-STING) reporter cell line (InvivoGen, US) was cultured according to the established protocol. HEK-STING cells were seeded into 96-well plates ($8\times10^4$ cells/well), and sEVs(N) or sEVs(H) were added to the cells. In the other three groups, sEVs(H) were pretreated with DNase, Sonication, or Sonication+DNase before being added. After incubation for the following 24 h, supernatants (50 μL) of each well were harvested and mixed with QUANTI-Blue™ solution (150 μL). The solution was incubated at 37 °C for 2 hours, and STING activation was determined by the value of absorbance (620 nm). The cells incubated with medium-only were considered as negative control.

*sEVs and allergic inflammation*

Human eosinophils were isolated from the peripheral blood of AR patients with a MACSxpress® eosinophil isolation kit (Stem Cell, Canada). The eosinophils were cultured in a specific medium with sEVs(N), sEVs(H), or sEVs(H)+C-178 and incubated at 37 °C for four hours. Eosinophils incubated with medium-only were considered as the Control group. Subsequently, DAPI staining and ECP immunostaining were applied and EETs in the slices were observed using CLSM.

The isolation and analysis of BMDCs from mouse bone marrow were performed according to the previous literature. Briefly, bone marrow was isolated from the femurs and tibias of mice, and the number of cells was adjusted to 2 × $10^6$ cells/mL at a 10 cm Petri dish. The cells were cultured in RPMI1640 medium containing 10% FBS (heat-inactivated), granulocyte-macrophage colony-stimulating factor (GM-CSF, 20 ng/mL) at 37 °C for 3 days. Subsequently, the culture medium in dishes was replaced by fresh RPMI1640 medium (10 mL), and the BMDCs were incubated for another 3 days. Finally, the medium containing suspended cells was changed, and BMDCs were harvested on day 9 for the following experiments.

BMDCs (5×$10^4$ cells/well) were seeded in a 12-well plate and incubated at 37 °C for 24 hours. Subsequently, the cells were treated with sEVs(N), sEVs(H), or sEVs(H)+C-178 for another 24 hours. sEVs were isolated from the medium of HDM-treated mice epithelial. The cells were harvested and washed with fluorescence-activated cell sorting (FACS) buffer. Finally, the cells were stained with CD45, CD11c, CD80, CD86, and MHC II antibodies and analyzed using flow cytometry (Beckman).

*Establishment of model mice with allergic airway inflammation*

A mouse model with allergic airway inflammation was established according to previous publications. BALB/c mice were intranasally sensitized by HDM (25 μg) in saline (50 μL) two times on the 0th and 7th day. Subsequently, the experimental mice were intranasally challenged with HDM (25 μg) in saline (50 μL) four times on the 14th, 15th, 16th, and 17th day. BALB/c mice sensitized and challenged with saline (same volume) were applied as sham mice.

*GW4869 and sEVs treatment for model mice*

Allergic model mice and sham mice were established according to the above protocol. In the GW+HDM group, GW4869 (10 μg) was locally administered at 8 h before the HDM challenge.

In the HDM+sEVs group, sEVs (10 μg) were intranasally administrated 4 hours after the HDM challenge, and sEVs were isolated from the medium of HDM-treated mice epithelial. The sham mice and allergic model mice treated with saline (50 μL) were considered negative controls and positive controls, respectively.

All of the experimental mice were sacrificed on the 18th day of the experiments, and the heads were quickly dissected to isolate the anterior part of the snout, including the nasal cavities. Subsequently, the maxillary bones (MBs) were decalcified in EDTA solution (10%) for 3 weeks before fixation in PFA (10%) for 24 hours. During the same time, lungs were isolated from the experimental mice and also fixed in PFA (10%) for 24 hours. Subsequently, the MBs and lungs were sequentially rinsed, dehydrated, and embedded in paraffin before the sections were cut and dewaxed. Finally, the slices were stained with IL-5 and ECP antibodies, and the positive staining area was analyzed by ImageJ software.

*Preparation of TP*

According to previous publications, $TiS_2$ monolayer nanosheets were produced using lithium anions as intercalation agents.[38] hPG (Mw ≈ 10,000 g/mol) was synthesized by one-pot, ring-opening anionic polymerization (ROAP), based on a reported method in the literature.[24,25] hPG with amino groups (PGA) was synthesized by a three-step protocol, including methylation, substitution, and reduction reactions.[25]

Lipoic acid (LA) solution (1 mL, 10 mg/mL) was mixed with $TiS_2$ solution (20 mL, 1 mg/mL) and then stirred at room temperature for 24 h to obtain LA-modified $TiS_2$ (TLA). Then, TLA (20 mg) was dispersed with EDC.HCl (1 mM) in MES solution (2 mL, 0.2 M), and the solution was then slowly added to PGA (2 g) in MES (20 mL, 0.2 M). The reaction was conducted for 2 d and then dialysis in Mili-Q water (MWCO=12–14 K) to obtain PGA-covered $TiS_2$ (TP).

*Synthesis of PNS and PNS$_E$*

HS-PEG-NHS (100 mg) was dissolved in PBS (100 mL) and then mixed with Glutamate (Glu, 50 mg) in PBS (1 mg/mL). After that, the mixture was dialysis (Mw=1,000) in MiliQ for 24 hours to obtain HS-PEG-Glu as crosslinking molecules. HS-PEG-Glu (100 mg) was added to TP solution (100 mL, 1 mg/mL) and stirred at room temperature for 24 h before dialysis (Mw=10,000) in MiliQ for 24 hours to obtain TP-Glu. Subsequently, TP-Glu (100 mg) with EDC.HCl (30 mg) was dispersed in MES (100 mL, 0.2 M). The mixture was dialysis (Mw=10,000) in MiliQ for 24 hours after stirring at room temperature for 24 hours. Finally, the crosslinked TP-Glu was incubated with horseradish peroxidase (HRP, 50 U/mL) and $H_2O_2$ (0.2 mM) at 37 ºC for 48 hours to obtain PG nanosheets (PNS).[39]

EGFR aptamer (10 mg) was added to PNS (200 mg) in MES (100 mL, 0.2 M) with EDC.HCl (60 mg) and stirred at room temperature for 24 hours. The mixture was dialyzed in MiliQ for 24 hours to obtain EGFR aptamer-modified PNS (PNS$_E$). EGFR aptamer-modified PG (PG$_E$) and TP (TP$_E$) were synthesized by similar protocols.

*Cytotoxicity test*

Human nasal epithelial (RPMI2650 cells) were cultured in Dulbecco's modified eagle's medium (DMEM) with 10% fetal bovine serum (FBS) and 1% penicillin-streptomycin (PS) at 37°C with 5% $CO_2$ in an incubator. The cytotoxicities of the PG, TP, PNS, PG$_E$, TP$_E$, PNS$_E$, and Apt$_E$ against RPMI2650 cells were measured with a CCK-8 assay. Firstly, RPMI2650 cells ($5\times10^3$ cells/well) were seeded in a 96-well plate and cultured until the cell density reached 60%. After that, the culture medium was replaced by PG, TP, PNS, PG$_E$, TP$_E$, PNS$_E$, and Apt$_E$ solution in DMEM with a series of concentrations (from 3 μg/mL to 1000 μg/mL). After 24 hours or 72 hours of incubation, the culture medium was replaced with fresh DMEM containing

10% CCK-8 reagent. Finally, the cells were incubated for another 2-3 hours at 37°C, and then the absorbance at 450 nm was measured using a Multiplate Reader. The cytotoxicities of PG, TP, PNS, $PG_E$, $TP_E$, $PNS_E$, and $Apt_E$ were calculated by the absorbance value compared to the cells treated with medium-only.

*Protein adsorption study*

BSA-FITC (100 μg/mL) was mixed with nanomaterials of different w/w ratios (1:1 and 1:2) and incubated at 37 °C for 30 min. Subsequently, the mixture was centrifuged at 11,000 rpm for 10 min, and the supernatant was collected to detect its fluorescence intensity. Protein adsorption (PA) was calculated as follows: PA = (C1 - C2) / C1 × 100%. C1 indicates the initial concentration of BSA, and C2 indicates the concentration of BSA in the supernatant after centrifugation.

*sEVs binding assay*

sEVs(H) were labeled with PKH67 dye and a standard curve for fluorescent intensity versus sEVs concentration was calculated. PKH67-labeled sEVs (1 μg/mL) were mixed with PG, TP, PNS, $PG_E$, $TP_E$, or $PNS_E$ in PBS (7.4) at different ratios (w/w = 1 or 2) and the solutions were incubated at 37 °C for 30 min. Finally, the supernatant was collected after centrifuging at 10,000 g (10 min), and the sEVs concentration was determined by fluorescence intensity (Ex=485 nm, Em=520 nm). The sEVs binding capacity was calculated based on the change of sEVs fluorescence in the supernatant. In another experiment, PNS, or $PNS_E$ were labeled with Cy5 dye and incubated with PKH67-labeled sEVs at 37 °C for 30 min. Then, the colocalization of sEVs and nanosheets was observed by CLSM (PKH67: 485 nm/520 nm; Cy5: 630 nm/670 nm) and quantitatively analyzed by ImageJ software.

HEK-STING cells were seeded into 96-well plates ($8 \times 10^4$ cells/well), and sEVs, sEVs+PG, sEVs+PNS, sEVs+$PG_E$, or sEVs+$PNS_E$ were added to the cells. sEVs were isolated from the medium of RPMI2650 cells treated with HDM (5 μg/mL). The concentration of sEVs and nanomaterials was set as 1 μg/mL and 2 μg/mL, respectively. After incubation for the following 24 hours, STING activation was determined by the value of absorbance (620 nm) with QUANTI-Blue™ assay. The cells incubated with medium-only or sEVs-only were considered as negative control and positive control, respectively.

*sEVs-induced EET formation*

Human eosinophils were isolated from the peripheral blood of AR patients with a MACSxpress® eosinophil isolation kit (Stem Cell, Canada). The eosinophils were cultured in a specific medium with sEVs, sEVs+PNS, sEVs+$PG_E$, or sEVs+$PNS_E$ and incubated at 37 °C for four hours. sEVs were isolated from the medium of HDM-treated RPMI2560 cells, and eosinophils incubated with medium-only were considered as the Control group. The concentration of sEVs and nanomaterials was set as 1 μg/mL and 2 μg/mL, respectively. Subsequently, DAPI staining and ECP immunostaining were applied to identify the nucleus and EETs. The EETs in slices were observed using CLSM and calculated by ImageJ software.

*Cellular uptake and co-localization of sEVs and nanosheets*

BMDCs ($2 \times 10^4$ cells/well) were seeded on the confocal slide inside a 24-well plate and incubated at 37 °C for 24 hours. Afterward, sEVs (PKH67-labeled, 1 μg/mL) and PNS, $PG_E$, or $PNS_E$ (Cy5-labeled, 5 μg/mL) were added, and the cells were incubated for another 24 hours. sEVs were isolated from the medium of HDM-treated mice epithelial, and the concentration of sEVs or nanomaterials was 1 μg/mL or 2 μg/mL, respectively. Subsequently, the supernatant was discarded, and cells were washed with PBS before fixation in PFA (4%) for 30 min. The

nucleus was then stained with DAPI for 20 min in the dark before the film was sealed with neutral gum, then the fluorescent images were observed by CLSM (DAPI: 360 nm/460 nm; PKH67: 488 nm/520 nm; Cy5: 630 nm/670 nm).

BMDCs ($2\times10^5$ cells/well) were seeded in a 12-well plate and incubated at 37°C overnight. Then the cells were treated with sEVs, sEVs+PNS, sEVs+$PG_E$, or sEVs+$PNS_E$, and incubated at 37°C for 24 hours. sEVs were labeled by PKH67, and the concentration was set at 5 μg/mL. Quantitative analysis of sEVs uptake was determined by monitoring the PKH67 fluorescence (488 nm/520 nm) signal by flow cytometry.

*sEVs-induced DC maturation*

BMDCs ($5\times10^4$ cells/well) were seeded in a 12-well plate and incubated at 37°C for 24 hours. Subsequently, the cells were treated with sEVs, sEVs+PNS, sEVs+$PG_E$, or sEVs+$PNS_E$ for another 24 hours. sEVs were isolated from the medium of HDM-treated mice epithelial, and the concentration of sEVs or nanomaterials was 1 μg/mL or 2 μg/mL, respectively. The cells were harvested, washed with FACS buffer, and stained with CD45, CD11c, CD80, CD86, and MHC II antibodies. Finally, the cells were analyzed for DC maturation using flow cytometry (Beckman), and the results were analyzed with FlowJo software.

*Determination of antioxidant capacity*

DPPH· solution (0.1 mM) was prepared with anhydrous ethanol for the following DPPH· reduction studies. Subsequently, PNS, $PG_E$, $TP_E$, and $PNS_E$ solution (1 mL) with a series of concentrations (1 μg/mL to 1000 μg/mL) was mixed with DPPH· solution (1 mL) and stirred at room temperature for 30 min. Finally, the solution was centrifugated at 5000 rpm for 10 min before the absorbance (517 nm) of the supernatant was measured. DPPH· mixed with an equal volume of MiliQ water was applied as the control group.

Salicylic acid solution (9 mM), $FeSO_4$ solution (9 mM), and $H_2O_2$ solution (8.8 mM) were prepared for ·OH clearance studies. Afterward, PNS, $PG_E$, $TP_E$, or $PNS_E$ solution (1 mL) with a series of concentrations (1 μg/mL to 1000 μg/mL) and the above three solutions (30 μL) were added to tubes. A negative control group without $H_2O_2$ and a positive control group without nanomaterials were established. All of the tubes were supplemented with MiliQ water to 450 μL, and the reactions were incubated at 37°C for 15 min before the absorbance (510 nm) was measured.

RPMI2650 cells were seeded into 96-well black plates ($5\times10^3$ cells/well) and stimulated with HDM (2 μg/mL) for 24 h. Subsequently, PG, PNS, $PG_E$, and $PNS_E$ (2 μg/mL) were added to the cells and incubated for another 3 hours before DCFH-DA was added. After 30 min, the fluorescent intensity of each well was measured by a Multiwell Platereader (495 nm/525 nm), and fluorescent images were recorded by CLSM.

*In vivo biosafety evaluation*

The in vivo biosafety of PNS, $PG_E$, $TP_E$, or $PNS_E$ was evaluated with healthy BALB/c mice (n=6). PNS, $PG_E$, $TP_E$, and $PNS_E$ (300 mg) were intranasally administered to the mice once per two days and four times in total. The mice treated only with saline were considered as a control group. Half of the mice were sacrificed on the 14th day, and the other half was sacrificed on the 28th day after the last treatment. MBs were collected from the experimental mice and fixed in PFA (4%) for 24 h before being decalcified in 10% EDTA at room temperature for 3 weeks. In addition, the hearts, livers, lungs, spleens, and kidneys of the mice were also collected and fixed in PFA (4%) for 24 h. After that, the decalcified MBs and organs were embedded in paraffin and H&E staining for tissue slices was employed for biosafety evaluation. In addition, the serum of experimental mice was collected, and alanine aminotransferase (ALT), aspartate

aminotransferase (AST), alkaline phosphatase (AKP), creatine kinase (CK), creatinine (CR), and CK-MB levels were determined to evaluate their hepatic and renal functions after treatment.

*Biodistribution of PNS, $PG_E$, and $PNS_E$*

Sham mice and allergic model mice were instilled (i.n.) with saline, PNS (20 μg), $PG_E$ (20 μg), or $PNS_E$ (20 μg) in saline (50 μL) 4 hours after the HDM challenge. After being raised for 1 day, 3 days, or 7 days, the experimental mice were sacrificed, then MBs, major organs (hearts, lungs, livers, spleens, and kidneys), and peripheral blood were harvested. The collected organs were imaged by an *in vivo* imaging system (630 nm/670 nm) to study the biodistribution of nanomaterials. The serum of mice was prepared, and the fluorescence intensity was measured to calculate the content of nanomaterials in the blood. In addition, frozen slices of MBs and lungs were prepared and analyzed by 3D imaging model of CLSM (DAPI: 360 nm/460 nm; Cy5: 630 nm/670 nm).

*Model mice treated with PNS, $PG_E$, and $PNS_E$*

Allergic model mice were established, and the mice were divided into the Saline group, HDM group, HDM+PNS group, HDM+$PG_E$ group, and HDM+$PNS_E$ group (n = 6). PNS (20 μg), $PG_E$ (20 μg), and $PNS_E$ (20 μg) in saline (50 μL) were instilled (i.n.) 4 hours after the HDM challenge, and the experimental mice were challenged and treated 4 times in total. One day after the last treatment, the experimental mice were sacrificed before NALF and BALF were collected. Subsequently, the fluids were sequentially centrifugated at 2000 g (10 min) and 10,000 g (30 min) to remove intact cells and cell debris. Then, sEVs concentration in fluids was determined by NTA, and the cytokine levels, including IL-4, IL-5, TNF-α, and TSLP, were measured with corresponding ELISA kits. In another experimental group, the nasal mucosa and lungs of mice were collected one day after the last treatment; then, the total mRNA was

extracted and converted to cDNA with commercial kits. Finally, Ct values for mRNA of cytokines (IL-4, IL-5, TSLP, and TNF-α) were determined by qRT-PCR, and the values were normalized to GAPDH. The gene sequences of each cytokine in the qRT-PCR experiments are listed in Table S1 of Supporting Information. In addition, the GSH, MDA, and SOD levels in the BALF of experimental mice were measured by the respective kits.

*Analysis of airway inflammation*

The experimental mice were sacrificed and decapitated 24 hours after the last treatment. Afterward, the MBs were collected after dissection to isolate the interior part of the snout and the nasal cavities. The MBs were decalcified in EDTA (10%) solution for 3 weeks after fixation in PFA (4%) for 24 hours. Subsequently, the specimens were carefully rinsed in water and dehydrated in dry ethanol. Finally, the specimens were embedded in paraffin, and the sections were cut, dewaxed, and stained by H&E. In addition, eosinophils staining, TB staining, ECP, and IL-5 immunostaining were also applied. The staining slices were observed by CLSM and analyzed by ImageJ software.

In addition to the nasal mucosa, the lungs of experimental mice were collected and fixed in PFA (4%) for 24 hours. Subsequently, the specimens were embedded in paraffin, and the sections were cut, dewaxed, and stained by H&E and PAS. Additionally, eosinophils staining, ECP, IL-5, CK-5, and CK-13 immunostaining were also applied to the slices. In another experimental group, the collected lung tissues were gently ground and filtered through a 40 μm cell sieve. Afterward, red blood cells (RBC) were removed using an RBC lysis solution to obtain a single-cell suspension. Subsequently, the cell types (including non-immune cells, T lymphocytes, neutrophils, eosinophils, DCs, macrophages, B lymphocytes, and other immune cells) were analyzed via flow cytometry using surface (or intracellular) markers.

*Transcriptome Sequencing*

TRIzol reagent was utilized for RNA extraction from lung tissues of the Saline group, HDM group, HDM+PNS group, HDM+$PG_E$ group, and HDM+$PNS_E$ group. Subsequently, the quality of the sample library was assessed using Agilent 2100 Bioanalyzer. Finally, the RNA sequencing of samples was analyzed using Next-Generation Sequencing (NGS) based on the Illumina sequencing platform.

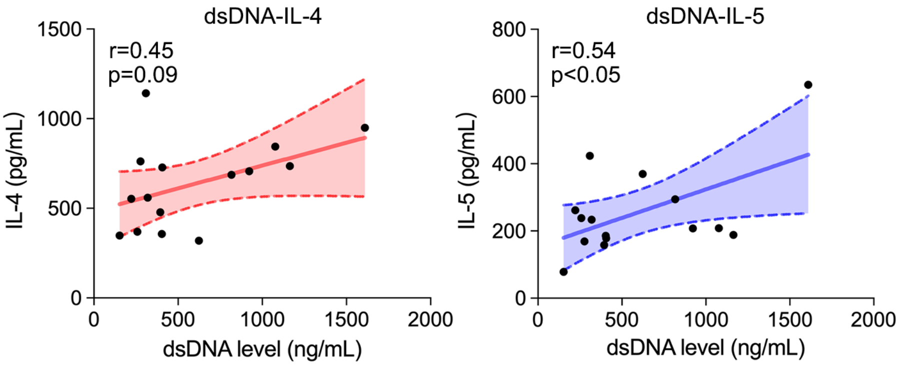


**Figure S1.** The correlations of IL-4 and IL-5 with dsDNA level in nasal secretions from AR patients.

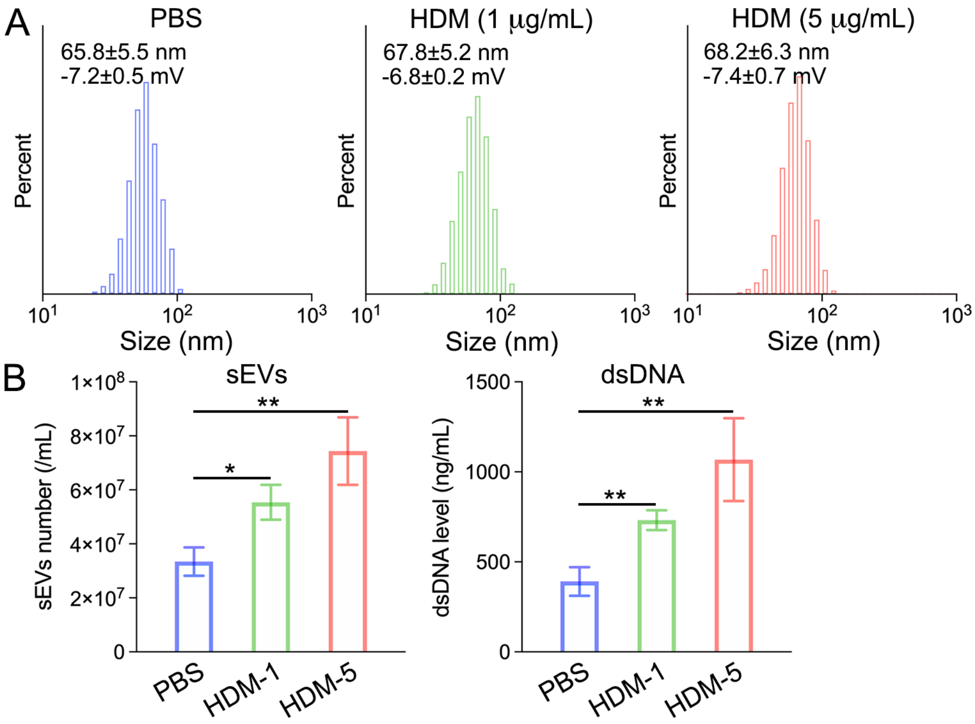


**Figure S2.** (A) The NTA results of sEVs isolated from the medium of mice epithelial treated with HDM. (B) The sEVs number and dsDNA level in the cell medium. Data represent mean ± S.D. (n=3, one-way ANOVA, ns represents no significant, $^{*}p < 0.05$, $^{**}p < 0.01$, $^{***}p < 0.001$).

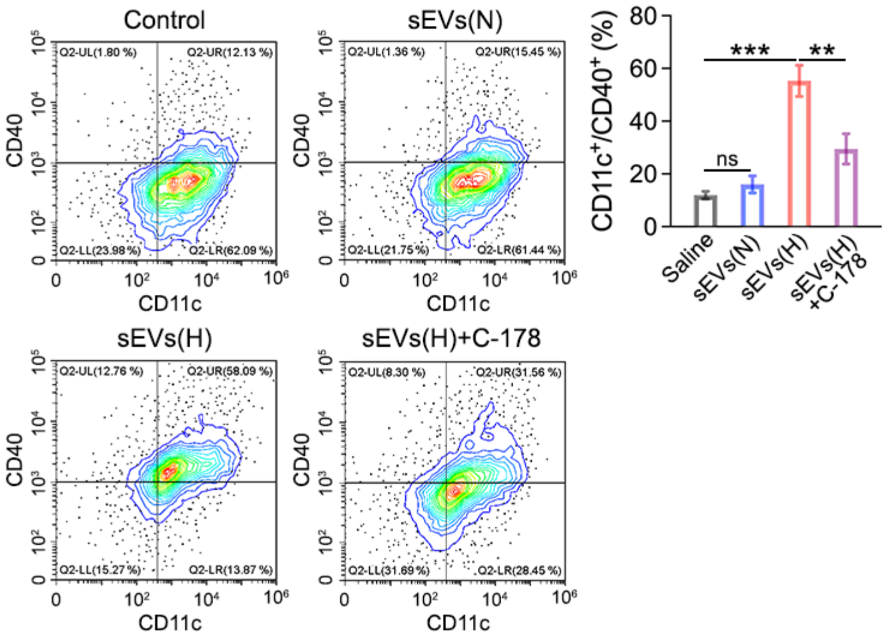


**Figure S3.** The representative flow cytometry dot plot of $CD11c^+/CD40^+$ BMDCs after incubation with sEVs(N), sEVs(H), and sEVs(H)+C-178. BMDCs incubated with medium only were considered as Control. Statistical analysis of the percentages of $CD11c^+/CD40^+$ BMDCs in different experimental groups. Data represent mean ± S.D. (n=3, one-way ANOVA, ns represents no significant, *$p < 0.05$, **$p < 0.01$, ***$p < 0.001$).

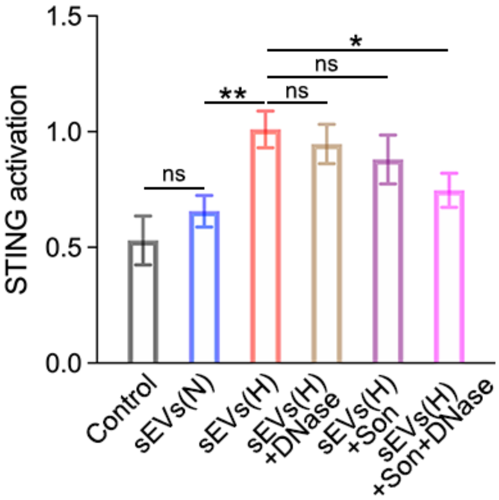


**Figure S4**. STING activation of HEK-STING cells induced by sEVs(N), sEVs(H), sEVs(H)+DNase, sEVs(H)+Son, and sEVs(H)+Son+DNase. Data represent the mean ± S.D. (n=3, one-way ANOVA,, ns represents no significance, *p<0.05, **p<0.01).

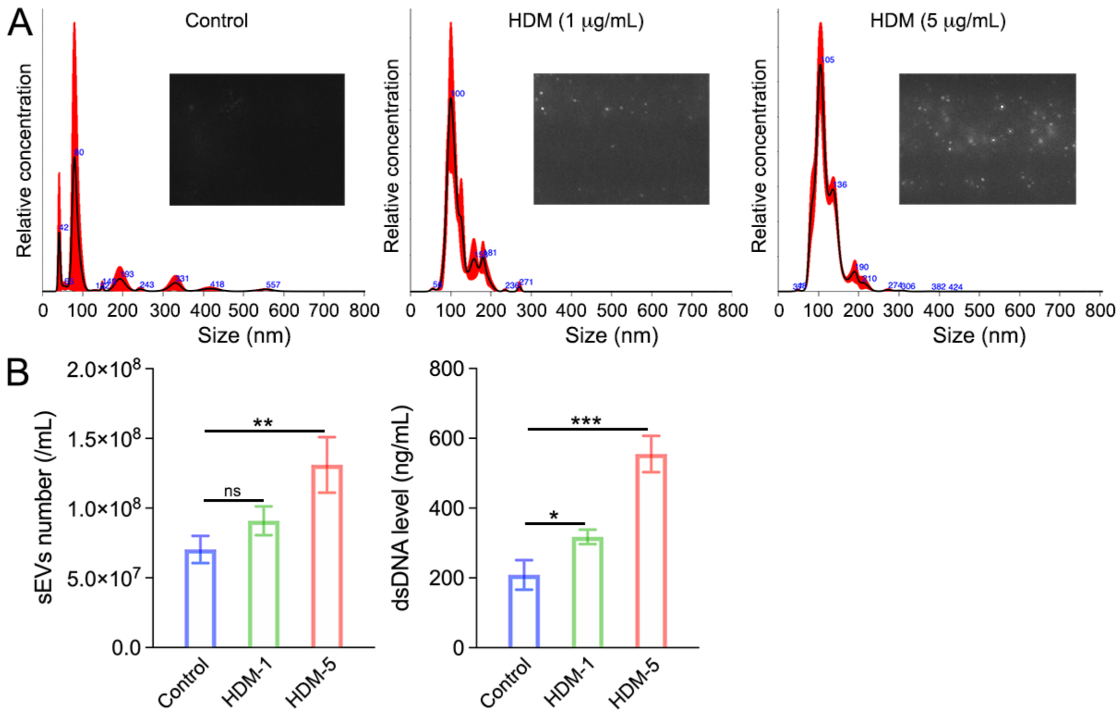


**Figure S5.** (A) The DLS and Zeta potential results of sEVs isolated from the medium of RPMI2650 cells treated with HDM. (B) The sEVs number and dsDNA level in the cell medium. Data represent mean ± S.D. (n=3, one-way ANOVA, ns represents no significance, *p < 0.05, **p < 0.01).

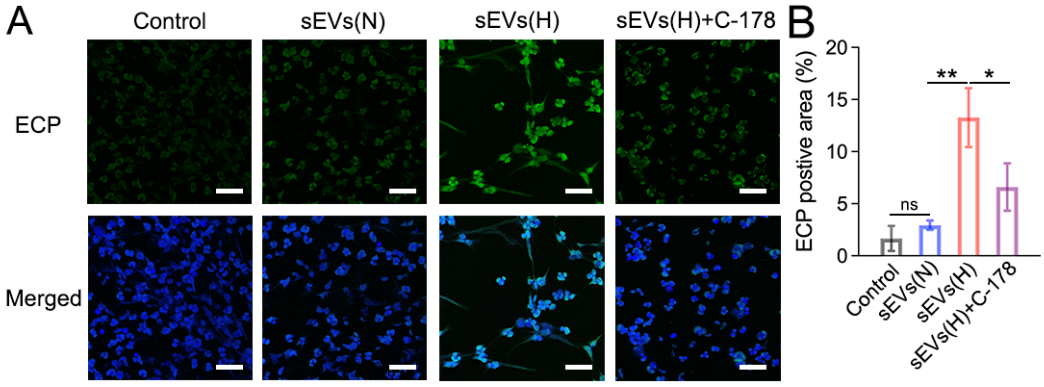


**Figure S6**. (A) Representative EET images of eosinophils treated with sEVs(N), sEVs(H), and sEVs(H)+C-178. Scale bars: 20 μm. (B) Quantification of the percentage of ECP-positive area in EET images. Data represent the mean ± S.D. (n=3, one-way ANOVA, ns represents no significance, *p<0.05, **p<0.01).

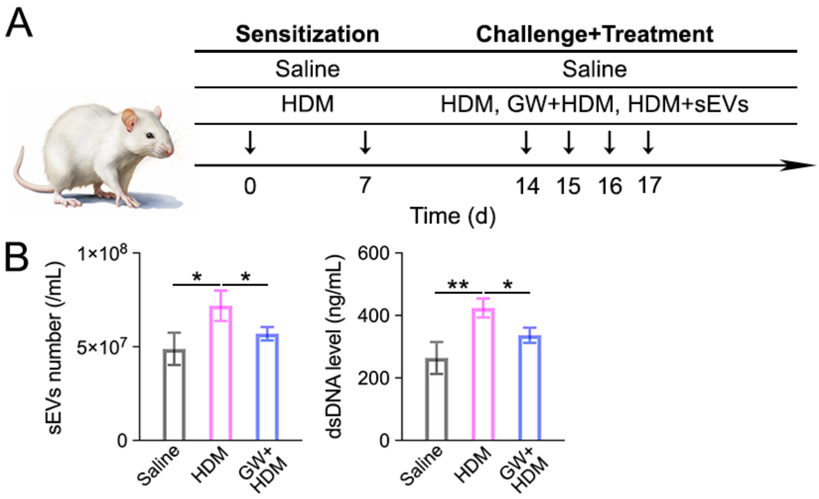


**Figure S7**. (A) Schematic diagram of the experimental protocol to establish the mouse model with allergic airway inflammation. (B) The sEVs number and dsDNA level in BALF of experimental mice. Data represent the mean ± S.D. (one-way ANOVA, *p<0.05, and **p<0.01).

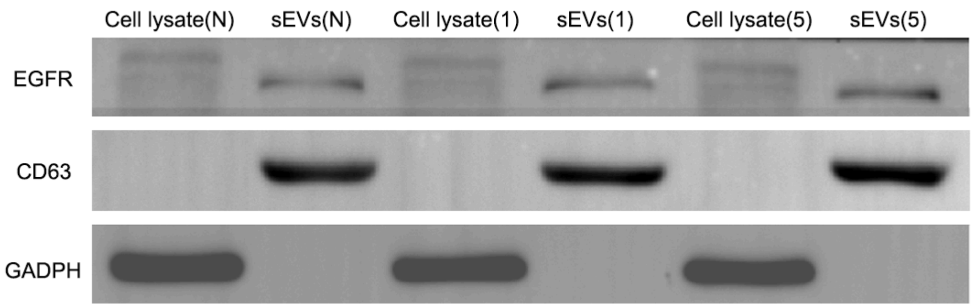


**Figure S8.** The WB images of cell lysate(N), sEVs(N), cell lysate(H-1), sEVs(H-1), cell lysate(H-5), and sEVs(H-5).

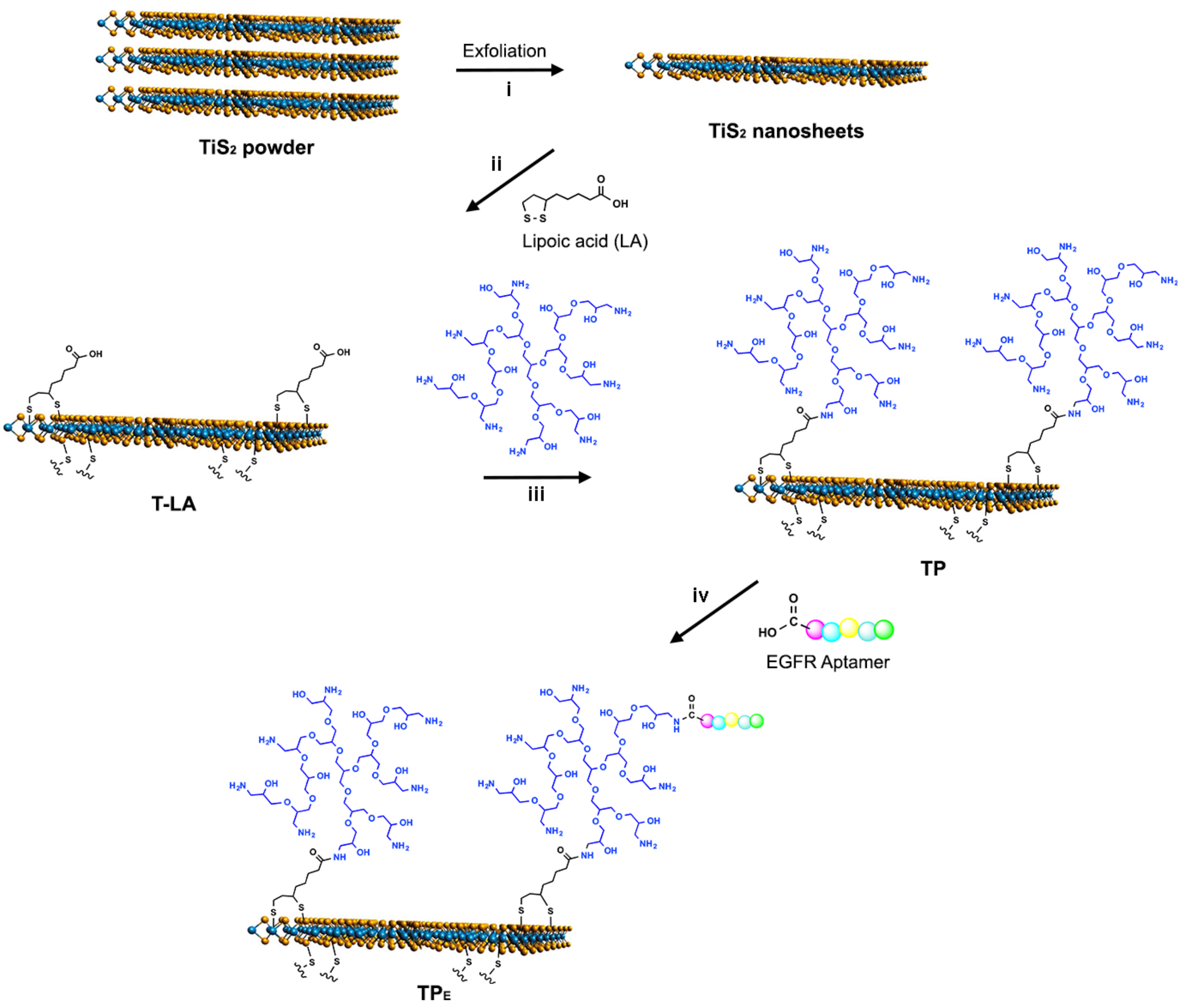


**Figure S9.** Synthesis routes of TP and $TP_E$. Detailed methods are provided in the Experimental section.

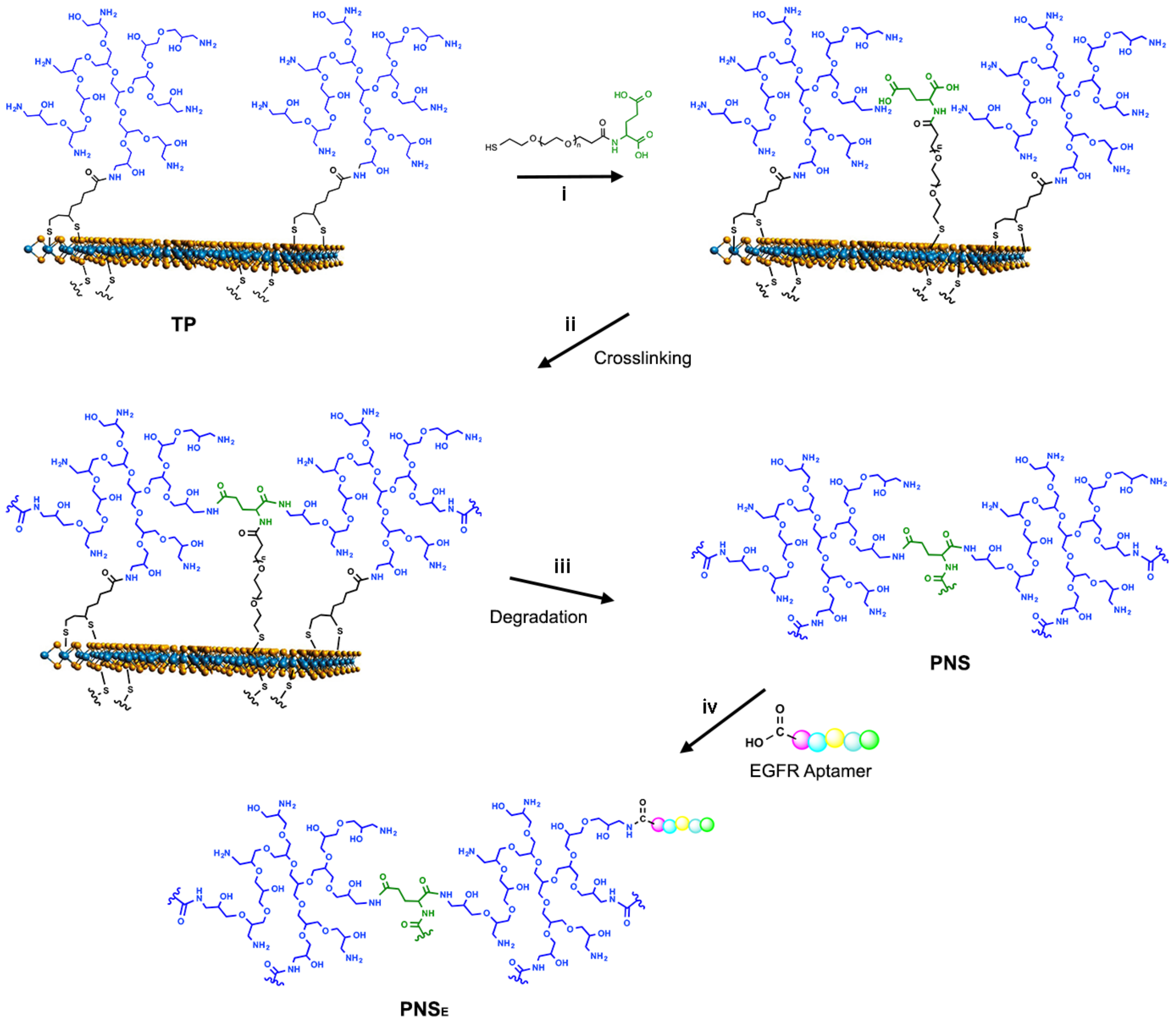


**Figure S10.** Synthesis routes for PNS and $PNS_E$. Detailed methods were provided in the Experimental section.

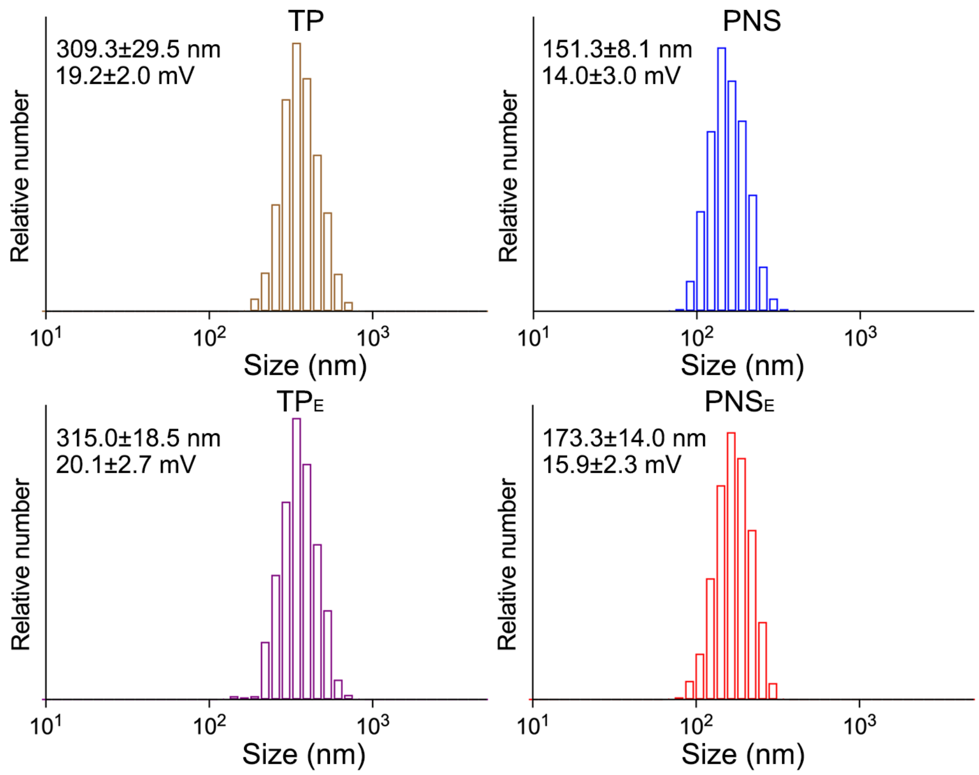


**Figure S11.** DLS and zeta potential results of TP, PNS, $TP_E$, and $PNS_E$ in PBS (7.4). Data represent mean ± S.D. (n=3).

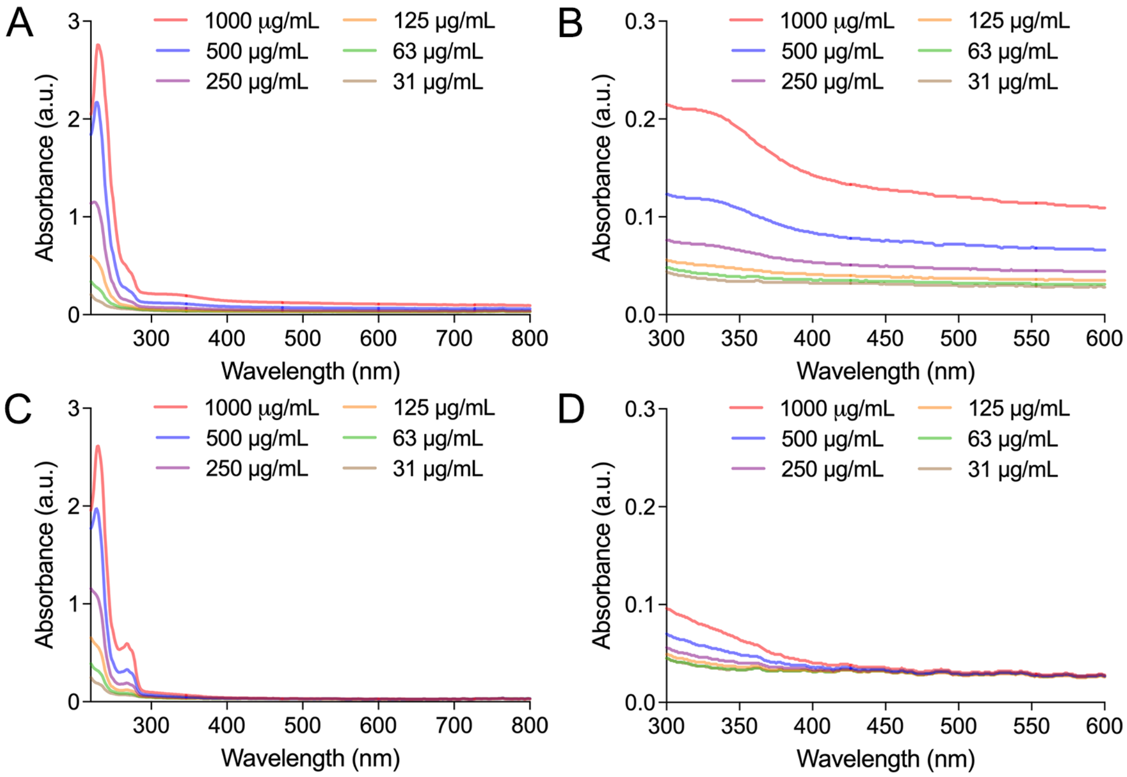


**Figure S12.** (A, B) UV–vis absorbance of TP at different concentrations from 31 to 1000 µg/mL. (C, D) UV–vis absorbance of PNS at different concentrations from 31 to 1000 µg/mL.

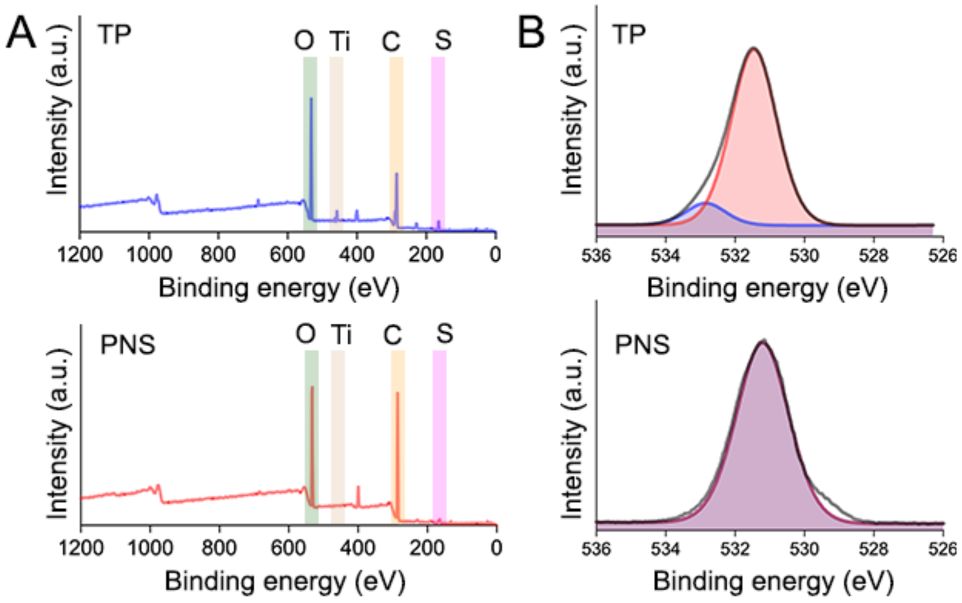


**Figure S13.** (A) XPS survey spectra of TP and PNS. (B) High-resolution XPS mapping of O 1s.

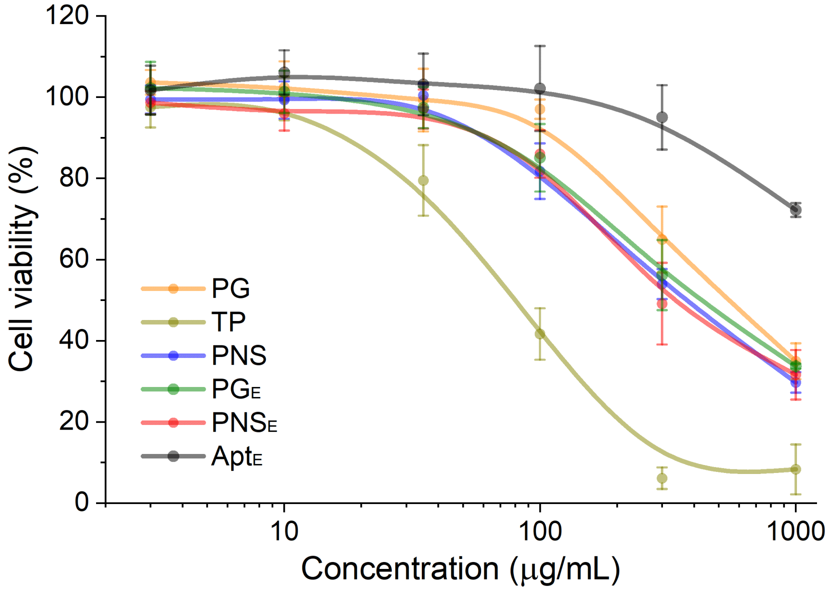


**Figure S14.** Biocompatibility of PG, TP, PNS, $PG_E$, $TP_E$, $PNS_E$, and $Apt_E$ against RPMI2650 cells for 72 h. Data indicate mean ± S.D. (n=3).

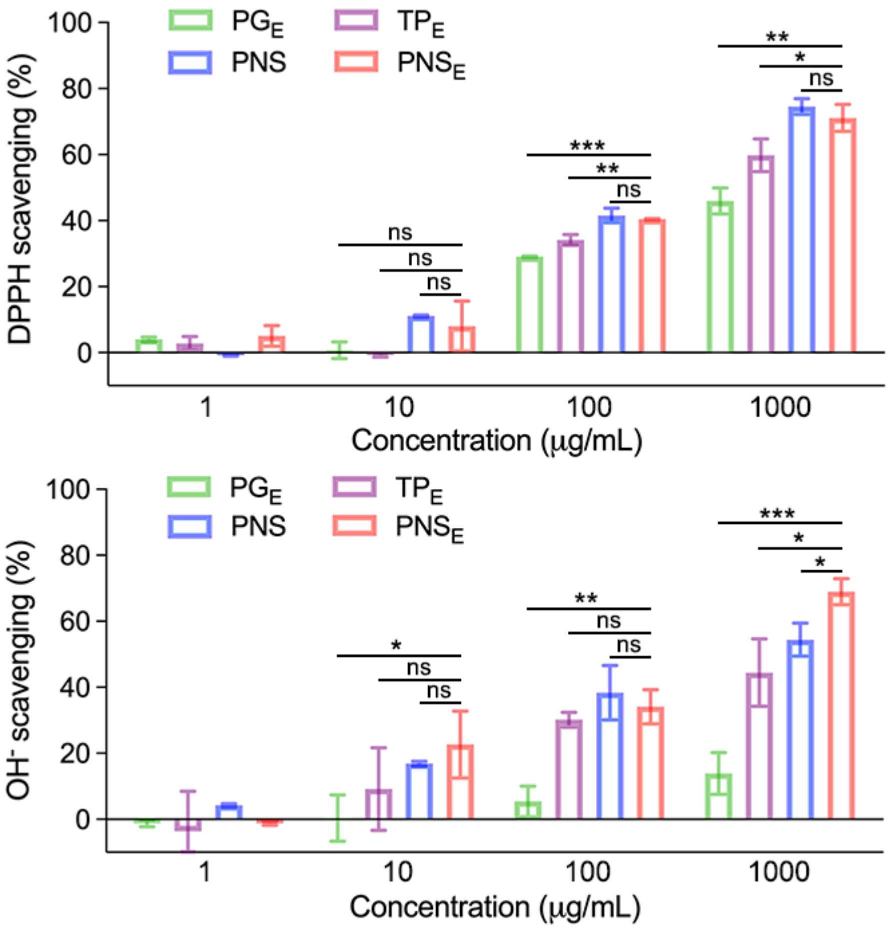


**Figure S15.** DPPH and $OH^-$ scavenging capacities of $PG_E$, $TP_E$, PNS, and $PNS_E$ in different concentrations. Data indicate mean ± S.D. (n=3, two-way ANOVA, ns represents no significant, *$p < 0.05$, **$p < 0.01$, ***$p < 0.001$).

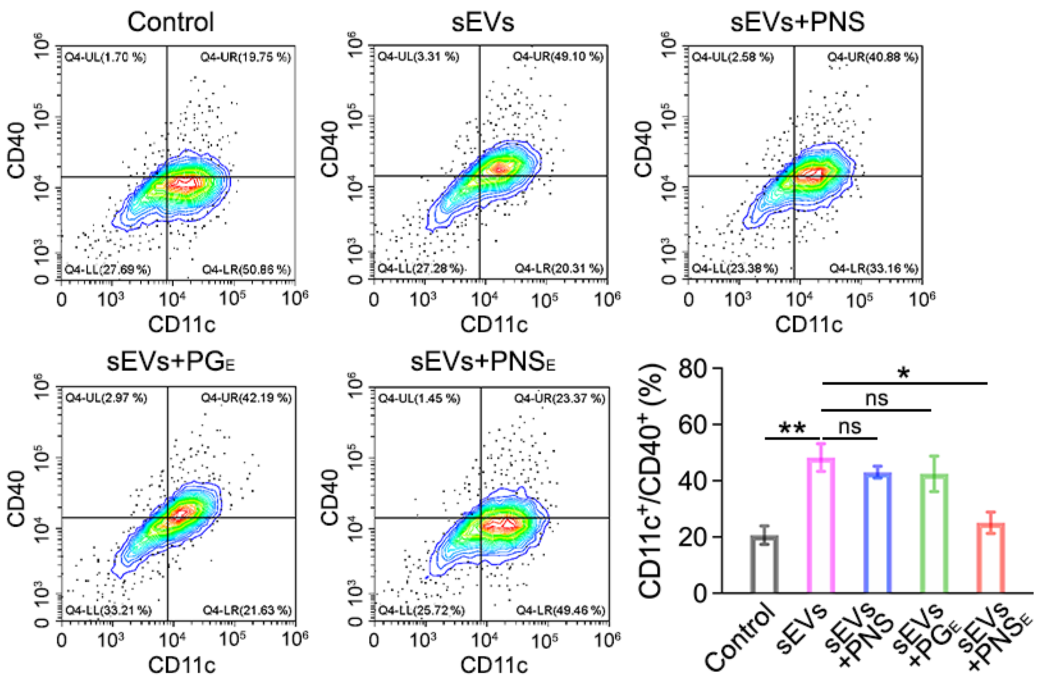


**Figure S16.** The representative flow cytometry dot plot of CD11c$^+$/CD40$^+$ BMDCs after incubation with sEVs, sEVs+PNS, sEVs+PG$_E$, and sEVs+PNS$_E$. BMDCs incubated with medium only were considered as Control. Statistical analysis of the percentages of CD11c$^+$/CD40$^+$ BMDCs in different experimental groups. Data represent mean ± S.D. (n=3, one-way ANOVA, ns represents no significance, *$p < 0.05$, **$p < 0.01$).

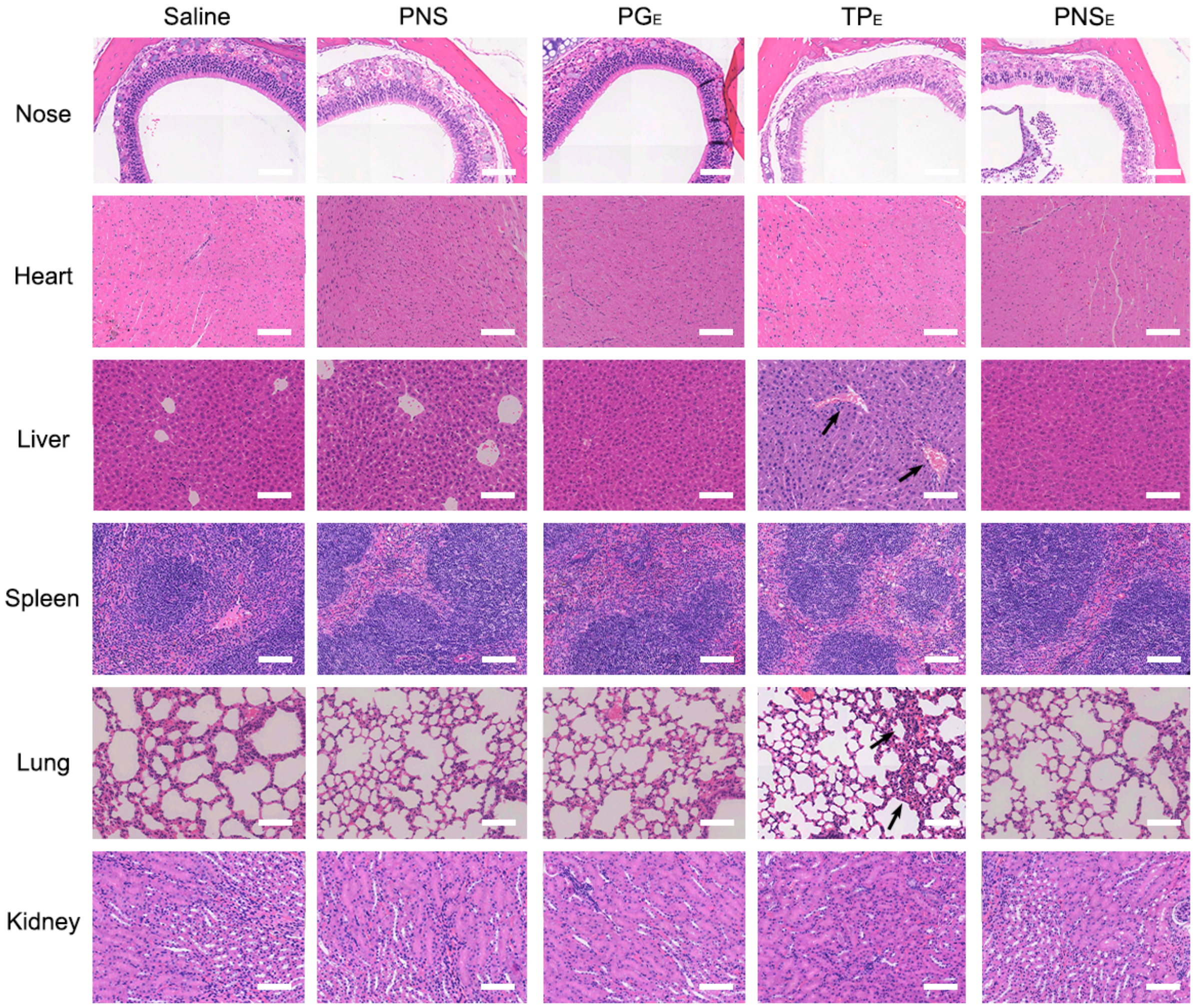


**Figure S17.** H&E staining of the nasal mucosa, hearts, livers, spleens, lungs, and kidneys from experimental mice after treatment with PNS, $PG_E$, $TP_E$, and $PNS_E$ for 14 days under light microscopy. Scale bars: 100 μm. Significant tissue damage could be observed in the livers and lungs of mice after treatment with $TP_E$.

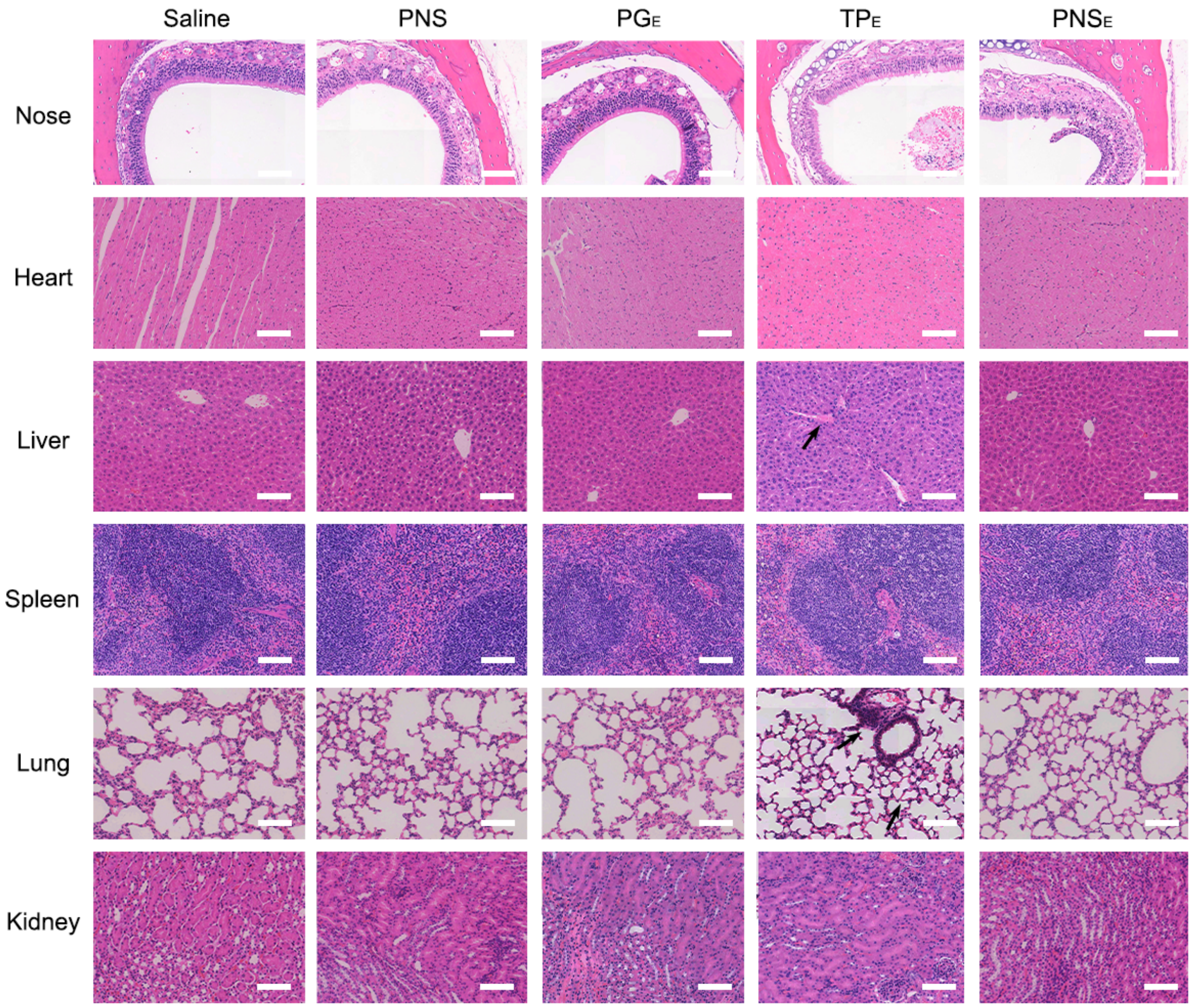


**Figure S18.** H&E staining of the nasal mucosa, hearts, livers, spleens, lungs, and kidneys from experimental mice after treatment with PNS, $PG_E$, $TP_E$, and $PNS_E$ for 28 days under light microscopy. Scale bars: 100 µm. Significant tissue damage could be observed in the livers and lungs of mice after treatment with $TP_E$.

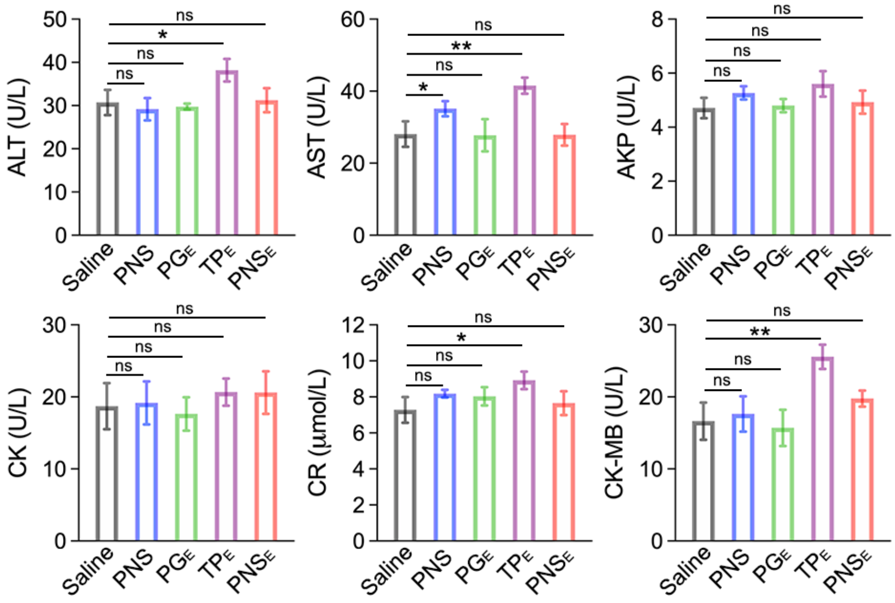


**Figure S19.** Biochemical parameters, including ALT, AST, AKP, CK, CR, and CK-MB of serum from experimental mice at 14th d after different treatments. Data represent mean ± S.D. (one-way ANOVA, ns represents no significance, *$p < 0.05$, **$p < 0.01$).

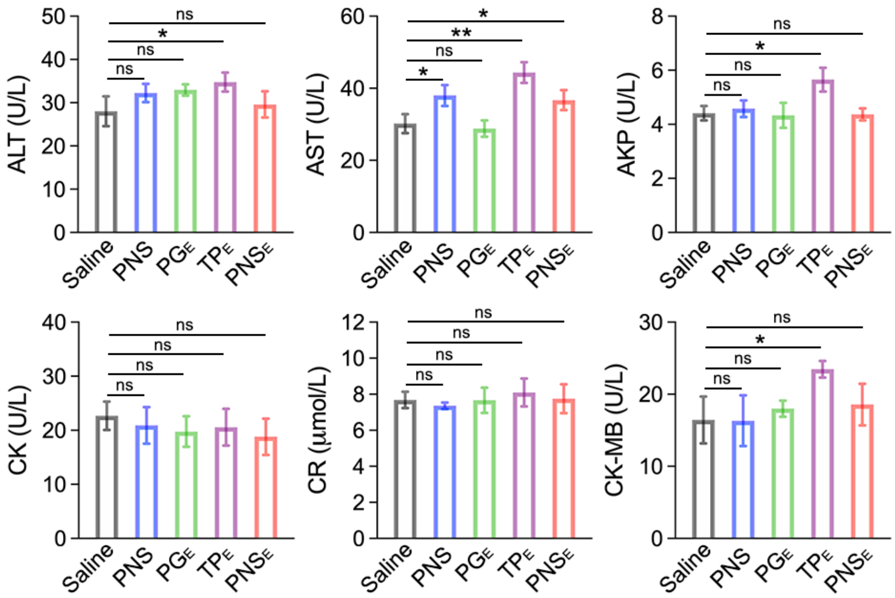


**Figure S20.** Biochemical parameters, including ALT, AST, AKP, CK, CR, and CK-MB of serum from experimental mice at 28th d after different treatments. Data represent mean ± S.D. (one-way ANOVA, ns represents no significance, *$p < 0.05$, **$p < 0.01$).

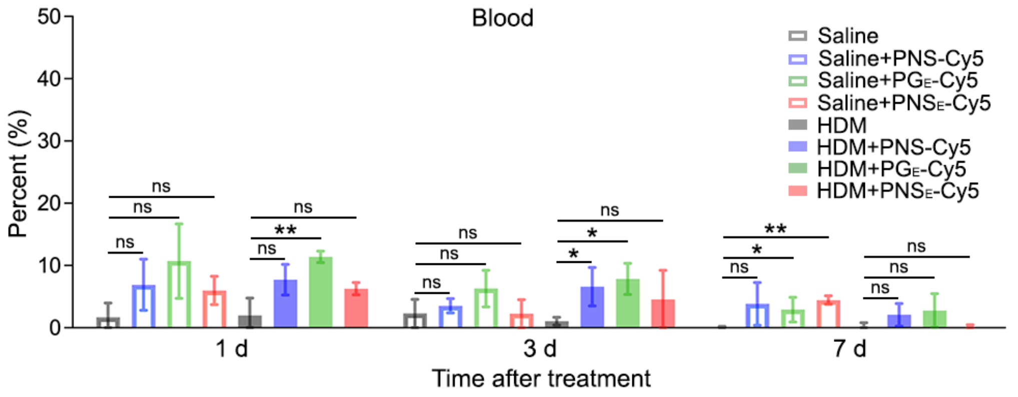


**Figure S21.** Quantification of fluorescence intensity in the blood of experimental mice after treatment with Cy5-labeled PNS, $PG_E$, and $PNS_E$. Data indicate mean ± S.D. (two-way ANOVA, ns represents no significance, $*p < 0.05$, $**p < 0.01$).

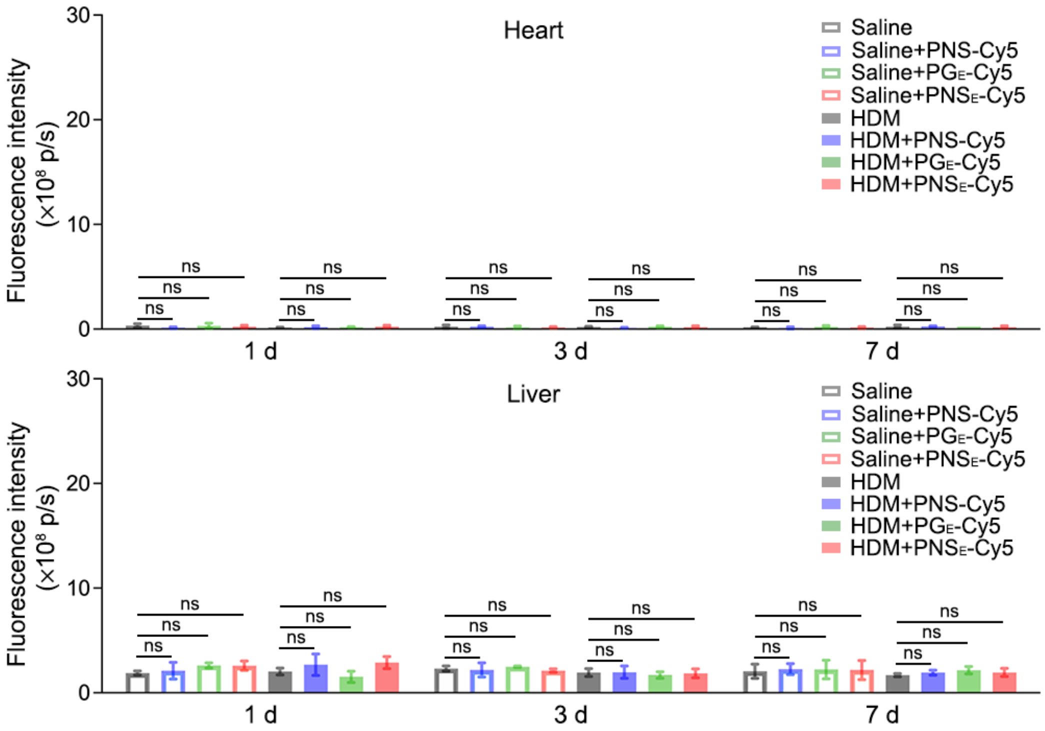


**Figure S22.** Quantification of fluorescence intensity in hearts and livers of experimental mice after treatment with Cy5-labeled PNS, $PG_E$, and $PNS_E$. Data indicate mean ± S.D. (two-way ANOVA, ns represents no significance).

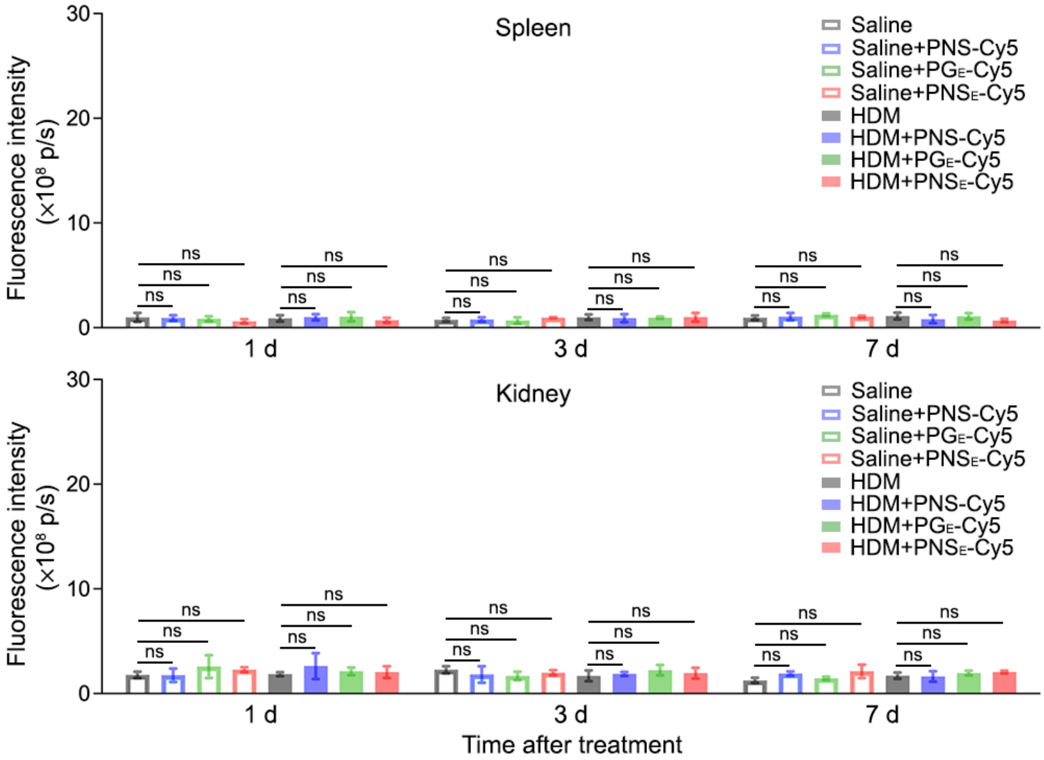


**Figure S23.** Quantification of fluorescence intensity in spleens and kidneys of experimental mice after treatment with Cy5-labeled PNS, $PG_E$, and $PNS_E$. Data indicate mean ± S.D. (two-way ANOVA, ns represents no significance).

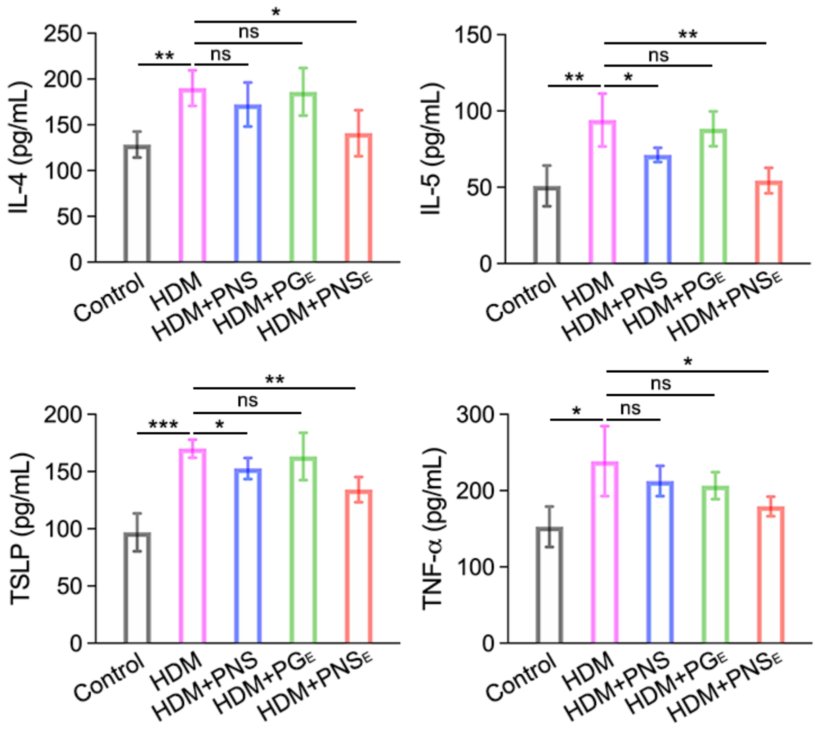


**Figure S24.** The IL-4, IL-5, TSLP, and TNF-α concentration in NALF of experimental mice. Data represent mean ± S.D. (one-way ANOVA, ns represents no significance, *p < 0.05, **p < 0.01, ***p < 0.001).

**Table S1.** Primer sequences in qRT-PCR experiments**.**

| Species | Target | Forward primer (5' to 3') | Reverse primer (5' to 3') |
|---|---|---|---|
| Mice | GAPDH | AGGTCGGTGTGAACGGATTTG | TGTAGACCATGTAGTTGAGGTCA |
| Mice | IL-4 | GGTCTCAACCCCCAGCTAGT | GCCGATGATCTCTCTCAAGTGAT |
| Mice | IL-5 | TCAGGGGCTAGACATACTGAAG | CCAAGGAACTCTTGCAGGTAAT |
| Mice | TSLP | CTTCTCAGGAGCCTCTTCA | AGCCAGGGATAGGATTGA |
| Mice | TNF-α | CAGGCGGTGCCTATGTCTC | CGATCACCCCGAAGTTCAGTAG |

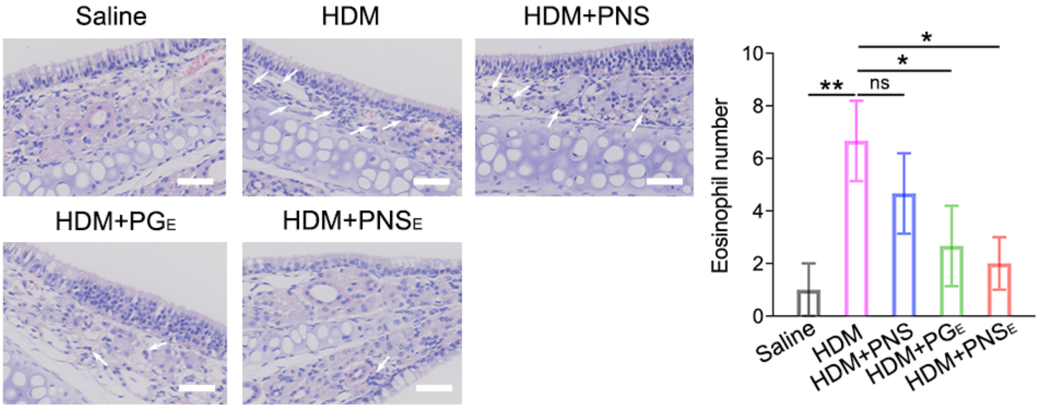


**Figure S25.** Representative eosinophil staining images of the nasal mucosa of experimental mice. Scale bars: 50 μm. Data represent mean ± S.D. (one-way ANOVA, ns represents no significance, *p < 0.05, **p < 0.01).

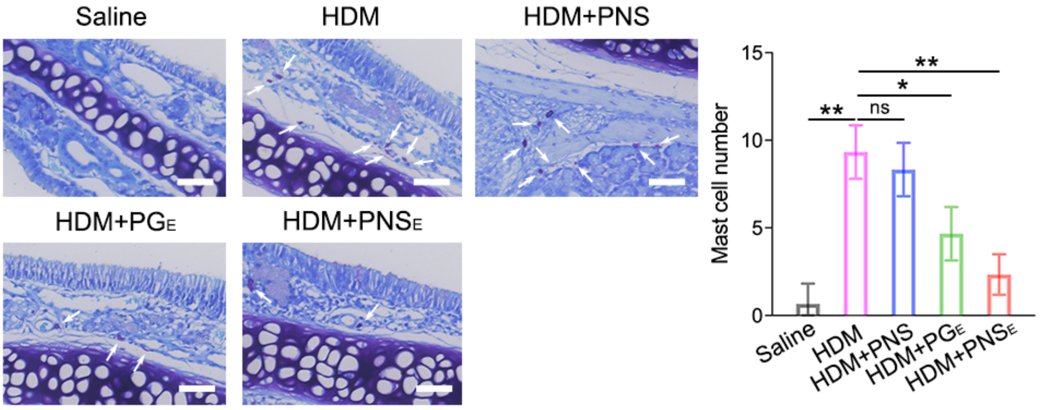


**Figure S26.** Representative TB staining images of the nasal mucosa of the experimental mice. Scale bars: 50 μm. Data represent mean ± S.D. (one-way ANOVA, ns represents no significance, *p < 0.05, **p < 0.01).

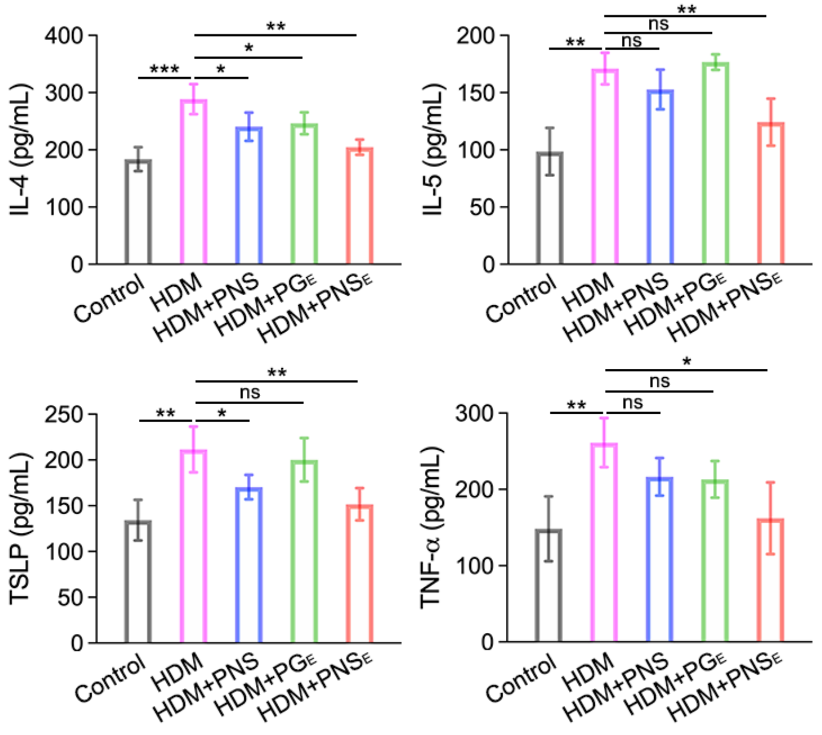


**Figure S27.** The IL-4, IL-5, TSLP, and TNF-α concentration in BALF of experimental mice in different treatment groups. Data represent mean ± S.D. (one-way ANOVA, ns represents no significance, *p < 0.05, **p < 0.01, ***p < 0.001).

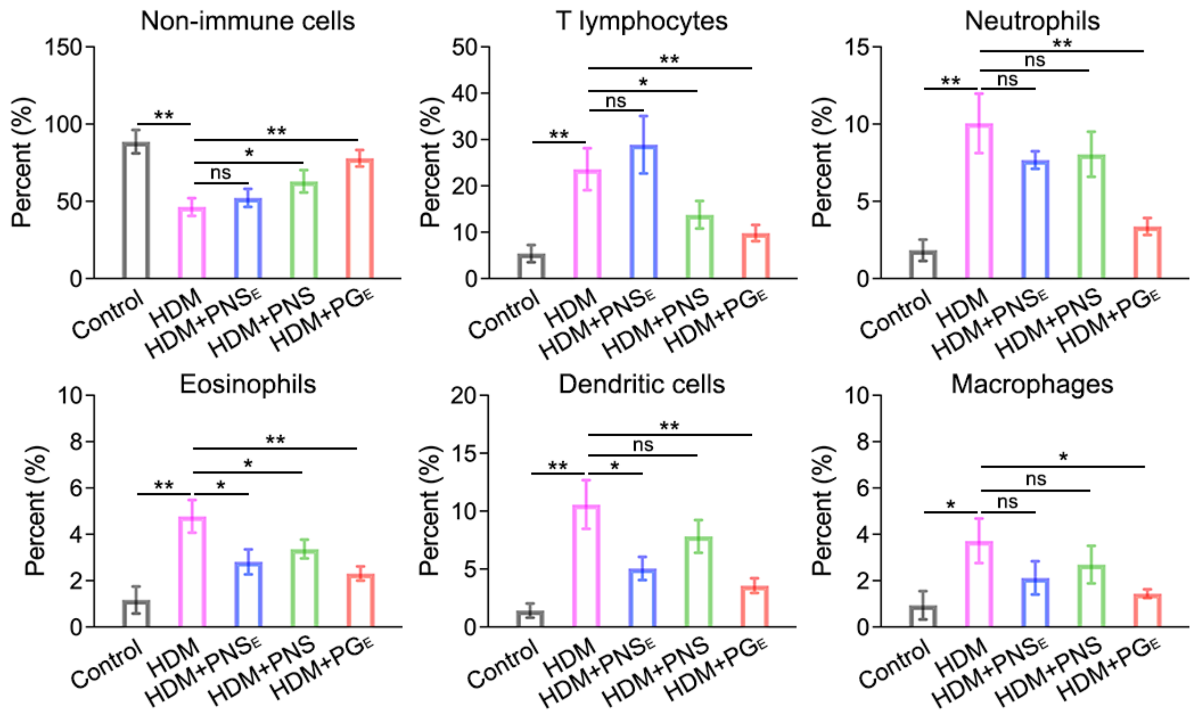


**Figure S28.** The percent of non-immune cells, T lymphocytes, neutrophils, eosinophils, DCs, and macrophages in the lungs of experimental mice in different treatment groups. The results were calculated based on the flow cytometry experiments. Data represent mean ± S.D. (one-way ANOVA, ns represents no significance, *p < 0.05, **p < 0.01).

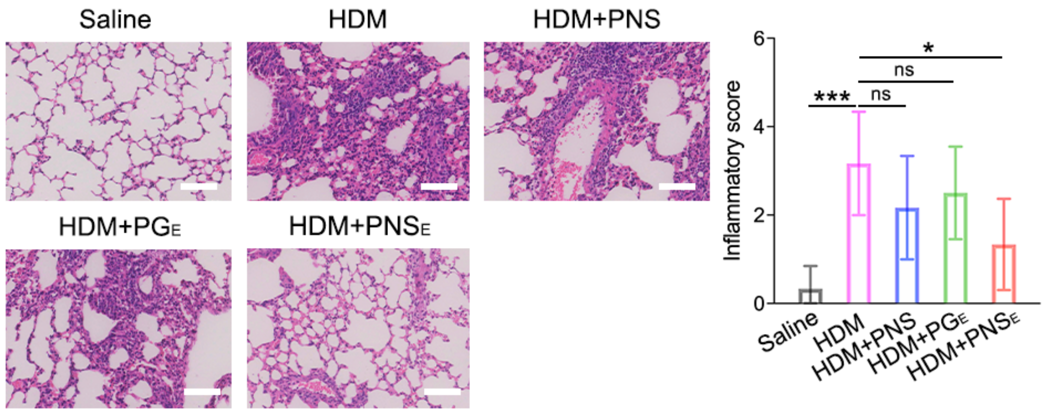


**Figure S29.** Representative H&E staining images of lungs from the experimental mice. Scale bars: 50 μm. Data represent mean ± S.D. (one-way ANOVA, ns represents no significance, *p < 0.05, **p < 0.01, ***p < 0.001).

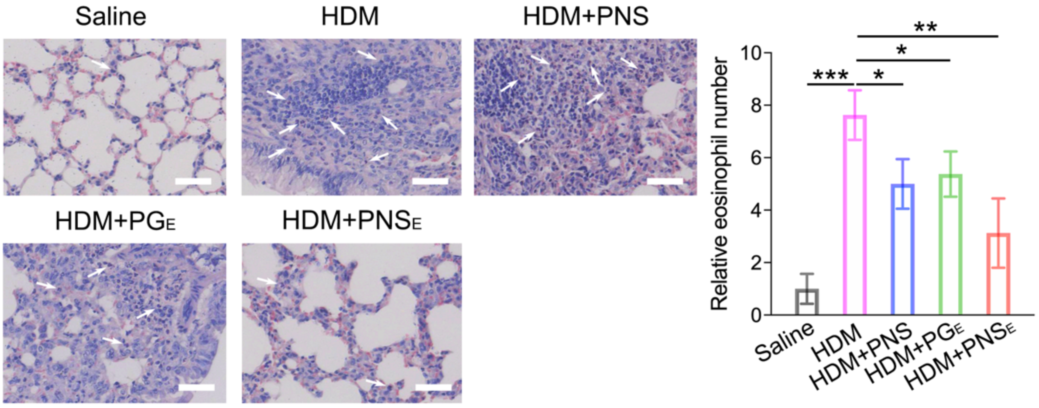


**Figure S30.** Representative eosinophil staining images of lungs from the experimental mice. Scale bars: 50 μm. Data represent mean ± S.D. (one-way ANOVA, *p < 0.05, **p < 0.01, ***p < 0.001).

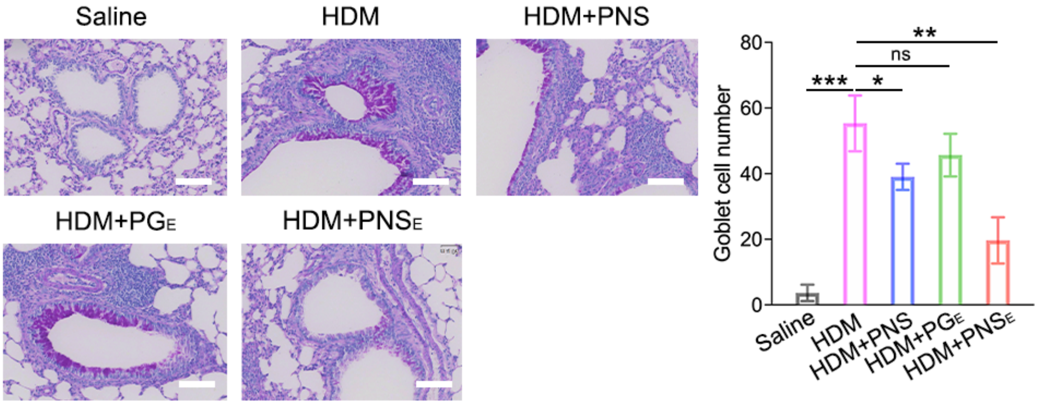


**Figure S31.** Representative PAS staining images of lungs from the experimental mice. Scale bars: 100 μm. Data represent mean ± S.D. (one-way ANOVA, ns represents no significance, *p < 0.05, **p < 0.01, ***p < 0.001).

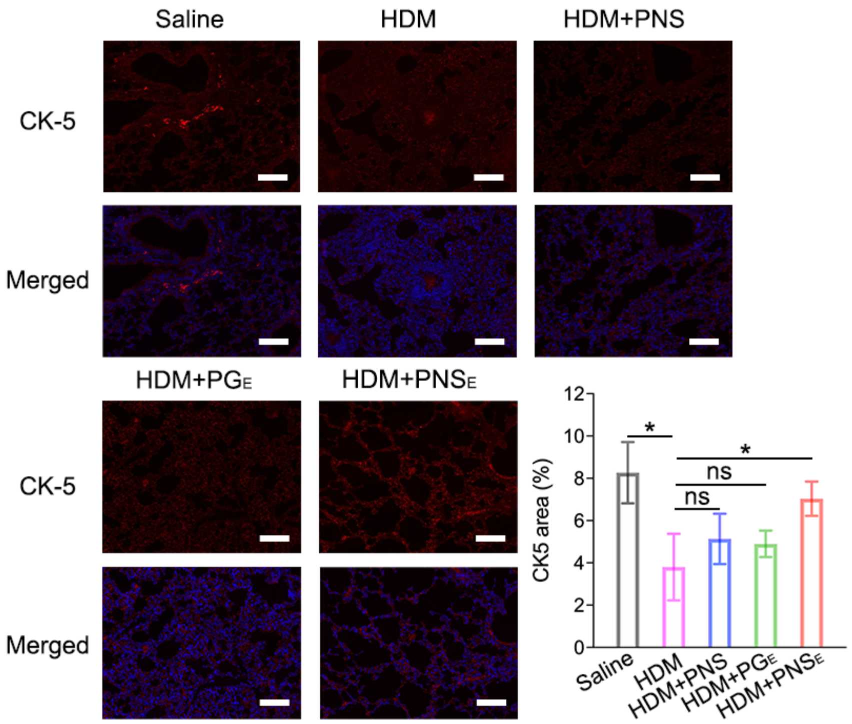


**Figure S32.** Representative CK-5 immunostaining images of lungs from the experimental mice. Scale bars: 100 µm. Data represent mean ± S.D. (one-way ANOVA, ns represents no significance, *$p < 0.05$).

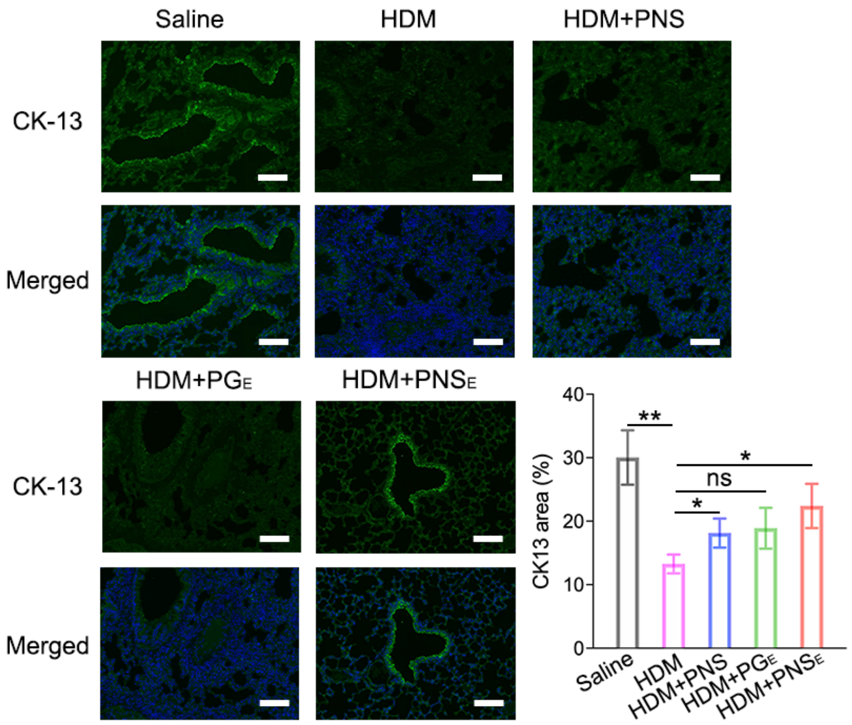


**Figure S33.** Representative CK-13 immunostaining images of lungs from the experimental mice. Scale bars: 100 μm. Data represent mean ± S.D. (one-way ANOVA, ns represents no significance, *p < 0.05, **p < 0.01).

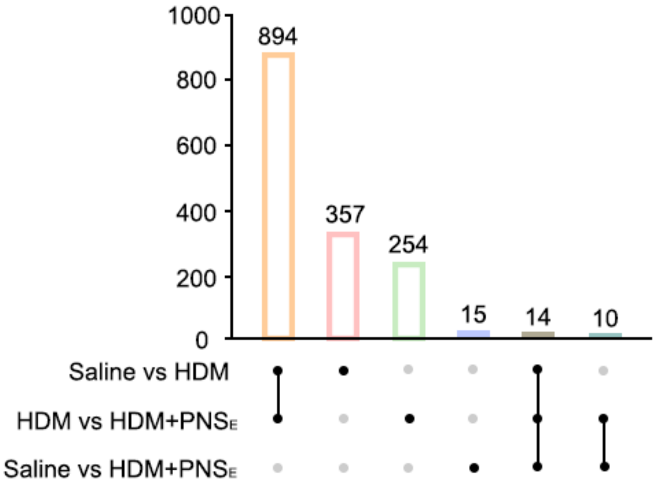


**Figure S34.** UpSet plot of DEGs between the Saline vs HDM group, HDM vs HDM+PNS$_E$ group, and Saline vs HDM+PNS$_E$ group.

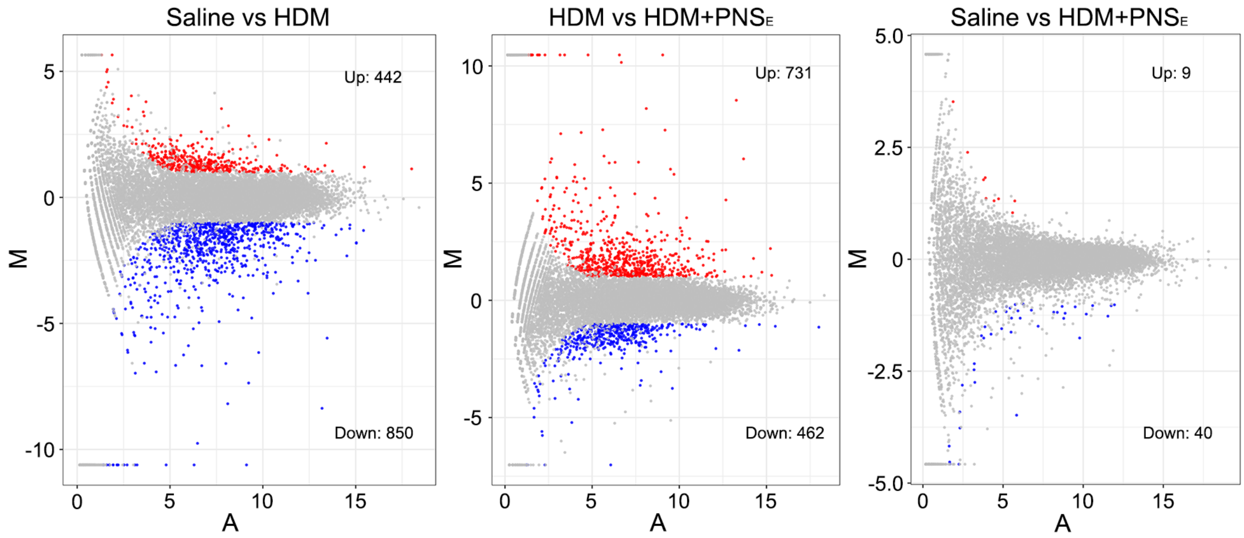


**Figure S35.** Minus-versus-Add (MA) maps based on the Saline vs HDM group, HDM vs HDM+PNS$_E$ group, and Saline vs HDM+PNS$_E$ group.

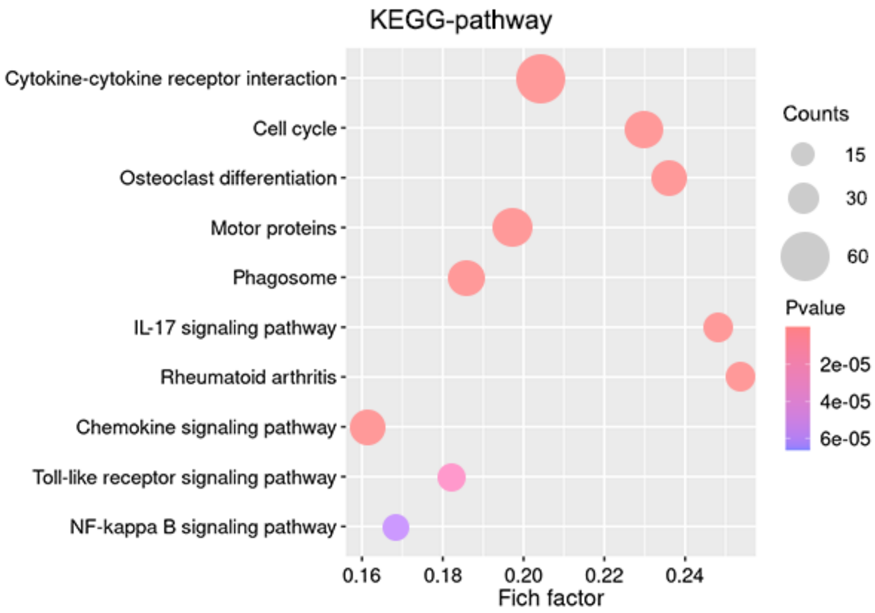


**Figure S36.** Kyoto Encyclopedia of Genes and Genomes (KEGG) pathway analyses of DEGs between the Saline vs HDM group.

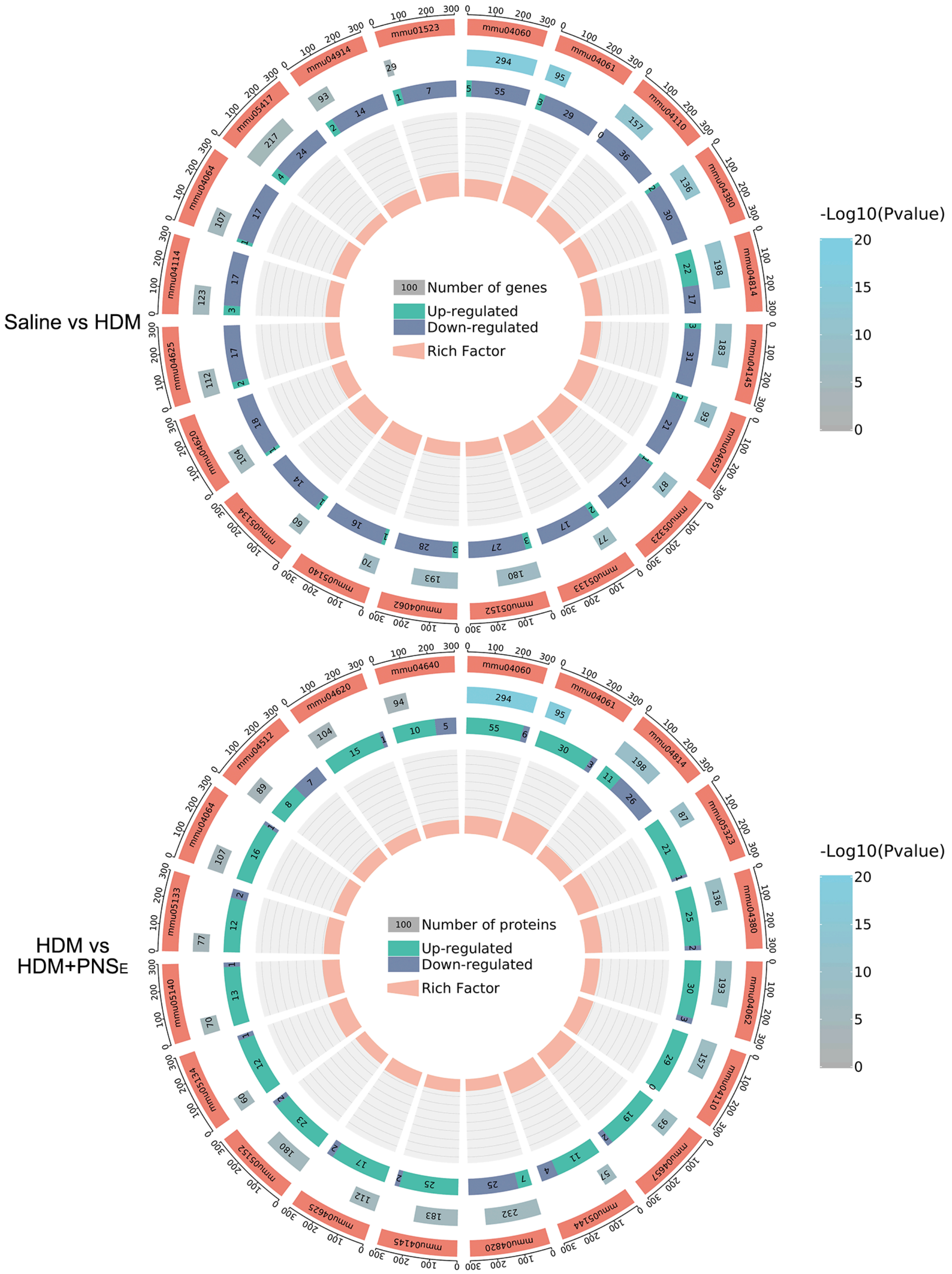


**Figure S37.** Reactome circle of KEGG enrichment entries of DEGs between Saline vs HDM and HDM vs HDM+PNSE group.

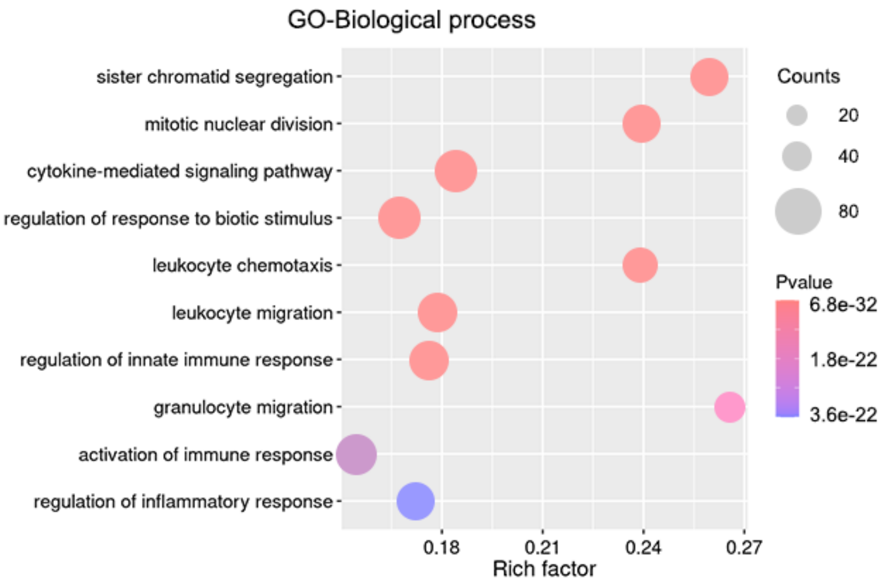


**Figure S38.** Gene Ontology (GO) term (biological process) enrichment analyses of DEGs between the Saline vs HDM group.

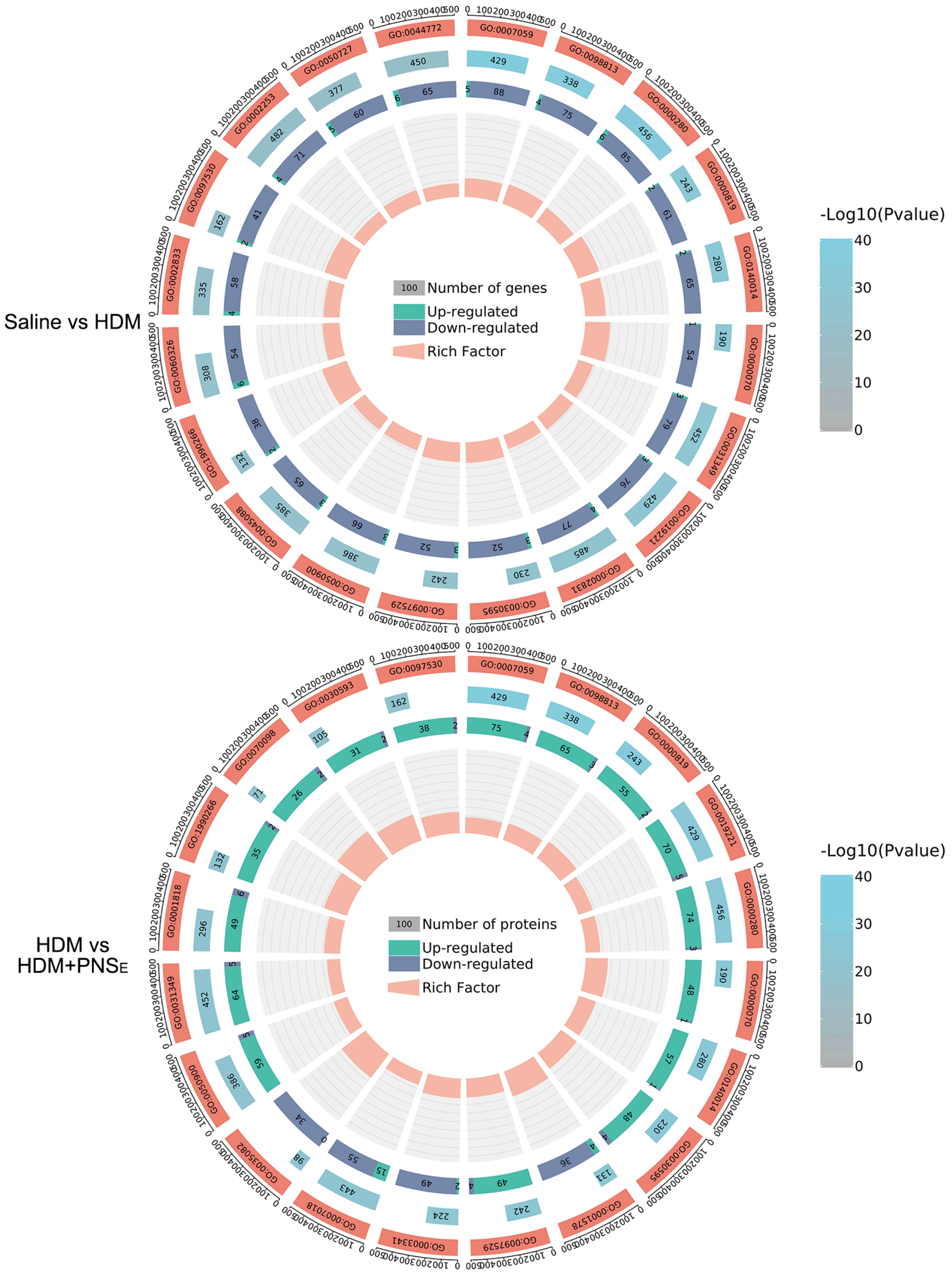


**Figure S39.** Reactome circle of GO enrichment entries of DEGs between Saline vs HDM and HDM vs HDM+PNSE group.
